# The fusion–fission optimization (FuFiO) algorithm

**DOI:** 10.1038/s41598-022-16498-4

**Published:** 2022-07-20

**Authors:** Behnaz Nouhi, Nima Darabi, Pooya Sareh, Hadi Bayazidi, Farhad Darabi, Siamak Talatahari

**Affiliations:** 1 Department of Mathematical Sciences, University of Tabriz, Tabriz, Iran; 2 Department of Civil, Constructional and Environmental Engineering, Sapienza University of Rome, Via Eudossiana, 18, 00184 Rome, Italy; 3 Creative Design Engineering Laboratory (Cdel), Department of Mechanical, Materials, and Aerospace Engineering, School of Engineering, University of Liverpool, Liverpool, L69 3GH UK; 4 Department of Civil Engineering, University of Tabriz, Tabriz, Iran; 5 Department of Physics, Azarbaijan Shahid Madani University, Tabriz, 53714-161 Iran; 6 Faculty of Engineering and Information Technology, University of Technology Sydney, Ultimo, NSW 2007 Australia

**Keywords:** Mathematics and computing, Physics

## Abstract

Fusion–Fission Optimization (FuFiO) is proposed as a new metaheuristic algorithm that simulates the tendency of nuclei to increase their binding energy and achieve higher levels of stability. In this algorithm, nuclei are divided into two groups, namely stable and unstable. Each nucleus can interact with other nuclei using three different types of nuclear reactions, including fusion, fission, and *β*-decay. These reactions establish the stabilization process of unstable nuclei through which they gradually turn into stable nuclei. A set of 120 mathematical benchmark test functions are selected to evaluate the performance of the proposed algorithm. The results of the FuFiO algorithm and its related non-parametric statistical tests are compared with those of other metaheuristic algorithms to make a valid judgment. Furthermore, as some highly-complicated problems, the test functions of two recent Competitions on Evolutionary Computation, namely CEC-2017 and CEC-2019, are solved and analyzed. The obtained results show that the FuFiO algorithm is superior to the other metaheuristic algorithms in most of the examined cases.

## Introduction

Optimization is a branch of applied mathematics that is widely used in various scientific disciplines because many problems can be expressed in the form of an optimization problem. Obviously, with the present rate of progress in all scientific fields, we face a variety of new real-world problems that have become more complex, such that conventional mathematical methods, such as exact optimizers, cannot solve them efficiently. In particular, exact optimizers do not have sufficient efficiency in dealing with many non-continuous, non-differentiable, and large-scale real-world multimodal problems^1^.

Early studies in the field of nature-inspired computation demonstrated that some numerical methods developed based on the behavior of natural creatures can solve real-world problems more effectively than exact methods^2^. Metaheuristic methods are numerical techniques that combine the heuristic rules of natural phenomena with a randomization process. Notably, over the past few decades, many researchers have concluded that developing and enhancing metaheuristic algorithms are practically-effective and computationally-efficient approaches to tackling complex and challenging unsolved real-world optimization problems^3–8^. A key advantage of metaheuristic methods is that they are *problem-independent* algorithms which provide acceptable solutions to complex and highly nonlinear problems in a reasonable time. Furthermore, they generally do not need any significant contributions to the algorithm structure from implementers, but it is only needed that they formulate the problem according to the requirements of the chosen metaheuristic. The point worth mentioning is that the core operation of the metaheuristic approaches is based on non-gradient procedures, where there is no need for cumbersome computations such as calculations of derivatives and multivariable generalizations. Moreover, randomization components enable metaheuristic algorithms to perform generally better than conventional methods. In particular, their stochastic nature enables them to escape from local optima and move toward global optimum on the search space of large-scale and challenging optimization problems.

Conventionally, two general criteria are used to classify metaheuristic methods: (1) the number of agents, and (2) the origin of inspiration. Based on the first criterion, metaheuristic algorithms can be divided into two groups: (1) single-solution-based algorithms, and (2) population-based algorithms. Also, according to inspiration, metaheuristic algorithms are divided into two main categories, namely Evolutionary Algorithms (EAs) and Swarm Intelligence (SI) algorithms. Single-solution-based methods try to modify one solution (agent) during the search process like what goes in the Simulated Annealing (SA) algorithm^9^; on the other hand, in population-based algorithms, a population of solutions is used to find the optimal answer similar to the simulation process in the Particle Swarm Optimization (PSO) algorithm^10^.

In EAs, the genetic evolution process is the main origin. Evolutionary Programming (EP)^2^, Evolutionary Strategy (ES)^11^, Genetic Algorithm (GA)^12^, and Differential Evolution (DE) are among the most famous methods in this domain. Besides, Simon^13^ proposed the Biogeography-Based Optimization (BBO) algorithm, which is used for global recombination and uniform crossover. Also, SI algorithms are based on the simulation of the collective behavior of creatures. SI algorithms are classified into three categories as follows. The first category is associated with the behavioral models of animals such as PSO^10^, Ant Colony Optimization (ACO)^14^, Artificial Bee Colony (ABC)^15^, Firefly Algorithm (FA)^16^, Cuckoo Search (CS)^17^, Bat Algorithm (BA)^18^, Eagle Strategy (ES)^19^, Krill Herd (KH)^20^, Flower Pollination Algorithm (FPA)^21^, Grey Wolf Optimizer (GWO)^22^, Ant Lion Optimizer (ALO)^23^, Grasshopper Optimization Algorithm (GOA)^24^, Symbiotic Organisms Search (SOS)^25,26^, Moth Flame Optimizer (MFO)^27^, Dragonfly Algorithm (DA)^28^, Salp Swarm Algorithm (SSA)^29^, Crow Search Algorithm (CSA)^30^, Whale Optimization Algorithm (WOA)^31,32^, Developed Swarm Optimizer (DSO)^33^, Spotted hyena optimizer (SHO)^34^, Farmland fertility algorithm (FFA)^35,36^, African Vultures Optimization (AVO)^37^, Bald Eagle Search Algorithm (BES)^38,39^ Tree Seed Algorithm (TSA)^40,41^, and Artificial Gorilla Troops (GTO) optimizer^42^. The second category concerns algorithms based on the physical and mathematical laws, such as Simulated Annealing (SA)^9^, Big Bang–Big Crunch optimization (BB–BC)^43^, Charged System Search (CSS)^44,45^, Chaos Game Optimization (CGO)^46,47^, Gravitational Search Algorithm (GSA)^48^, Sine Cosine Algorithm (SCA)^49^, Multi-Verse Optimizer (MVO)^50^, Atom Search Optimization (ASO)^51^, Crystal Structure Algorithm (CryStAl)^52–55^, and Electromagnetic field optimization (EFO)^56^. The third category includes algorithms that mimic various optimal behaviors of humans, for example, Imperialist Competitive Algorithm (ICA)^57^, Teaching Learning Based Optimization (TLBO)^58^, Interior Search Algorithm (ISA)^59^, and Stochastic Paint Optimizer (SPO)^60^.

Though there is a wide range of metaheuristic methods developed over the past few decades, they solve problems with different accuracies and time efficiencies; that is, one algorithm may not solve a specific problem with a desired accuracy or within a reasonable time, whereas another algorithm may be capable of achieving this goal. Therefore, computational time and accuracy are two essential considerations in developing novel metaheuristic methods. In other words, new robust methods are developed for more efficient search in the space of problems, and to find more accurate solutions to complex and large-scale problems in less time than previous ones. Therefore, there is an ongoing ambition in the optimization community to develop novel high-performance optimizers which can solve challenging problems more efficiently. In other words, each algorithm has particular advantages and disadvantages that are listed in Table 1 for the abovementioned algorithms.

**Table 1:** Advantages and disadvantages of various metaheuristic algorithms.

Algorithm	References	Advantages	Disadvantages
GA	^61^	Simplicity, flexibility, and ease of implementation Ability to deal with complex fitness landscapes	Slow convergence rate Having several tuning parameters Getting easily stuck in local optima
DE	^62^	Simplicity, flexibility, and ease of implementation Robustness	Having several tuning parameters Getting easily stuck in local optima
BBO	^63^	Simplicity, flexibility, and ease of implementation	Slow convergence rate Having several tuning parameters Low exploration capability
PSO	^64^	Simplicity, flexibility, and ease of implementation	Getting easily stuck in local optima High sensitivity to parameters tunning
ACO	^65^	Suitability for discrete and combinatorial problems Satisfying the local and global searches of the entire search space	Not suitable for continuous problems Getting easily stuck in local optima High computational cost
ABC	^66^	Simplicity, flexibility, and ease of implementation Good exploration capability Having only one parameter to be tunned	Slow convergence rate Low exploitation capability Getting easily stuck in local optima
FA	^67^	Simplicity, flexibility, and ease of implementation Being a memory-less algorithm	Slow convergence rate Having several tuning parameters Low exploration capability
CS	^68^	Simplicity, flexibility, and ease of implementation Having only one parameter to be tunned	Slow convergence rate Getting easily stuck in local optima
BA	^69^	Simplicity, flexibility, ease of implementation	Fast convergence in early iterations and subsequent slow-down Having several tuning parameters Getting easily stuck in local optima
Eagle Strategy	^19^	Efficiency in exploration and exploitation	Having several tuning parameters Getting easily stuck in local optima
KH	^70^	Ease of implementation Having only one parameter to be tunned	Slow convergence rate Getting easily stuck in local optima
FPA	^71^	Simplicity, flexibility, and ease of implementation	Suffering from premature convergence Having several tuning parameters Being time-consuming
GWO	^72^	No need for a larger storage Fast convergence	Getting trapped in local optima of large-scale problems
ALO	^73^	High feasibility and efficiency in reaching global optima	Suffering from premature convergence Probability distribution changes by generations Relatively not simple
GOA	^74^	Simplicity, flexibility, and ease of implementation	Slow convergence rate Getting easily stuck in local optima
SOS	^75^	Being a parameter-free algorithm Satisfying the local and global searches of the entire search space Good exploitation capability	Low computational efficiency Poor performance in handling high-dimensional and complex problems
MFO	^76^	Simplicity, flexibility, and ease of implementation	Slow convergence rate Getting easily stuck in local optima Having several tuning parameters
DA	^77^	Powerful neighborhood search characteristics Easy to merge with other algorithms	Suffering from premature convergence Getting easily stuck in local optima Having several tuning parameters
SSA	^78^	Few control parameters High feasibility and efficiency in reaching global optima	Suffering from premature convergence Probability distribution changes by generations
CSA	^79^	Simplicity, flexibility, and ease of implementation Few control parameters	Slow convergence rate Getting easily stuck in local optima Poor performance in handling high-dimensional and complex problems
WOA	^80^	Appropriate convergence rate Powerful neighborhood exploration characteristics Lower probably of trapping into local optima	Several tuning parameters May suffer from premature convergence Probability distribution changes by generations
DSO	^33^	Effectively avoiding local optimality with a non-increasing uncertainty	Several tuning parameters High computational time
SHO	^81^	Simplicity, flexibility, and ease of implementation Compatibility, robustness, and scalability	Suffers from premature convergence Proneness to get stuck in local optimums Long iterations in some problems
FFA	^35^	Appropriate convergence rate	Relatively high computational cost Several tuning parameters
AVO	^37^	Good convergence performance in handling some complex optimization problems Performing well in high-dimensional problems	Relatively complex Several tuning parameters
BES	^38^	Simplicity, flexibility, and ease of implementation Appropriate balance between exploration and exploitation abilities	May stuck in local optimums Several tuning parameters
TSA	^82^	Simplicity, flexibility, and ease of implementation Has just one parameter to be tunned	May stuck in local optimums Low effectiveness in solving complex and high dimensional optimization problems
GTO	^42^	Compatibility, robustness, and scalability Good convergence performance in handling some complex optimization problems	Relatively complex Several tuning parameters Relatively high computational cost
SA	^83^	Simplicity and ease of implementation Sound theoretical guarantees	Getting easily stuck in local optima Long computational time Sensitivity to parameters tunning
BB–BC	^84^	Simplicity and ease of implementation Few control parameters	Suffering from premature convergence Easily getting stuck in local optima
CSS	^85^	Simplicity and ease of implementation Efficiency for engineering applications	Several tuning parameters May get stuck in local optima Relatively high computational cost
CGO	^47^	Being a parameter-free algorithm Appropriate convergence rate Satisfying the local and global searches of the entire search space	May get stuck in local optima for special problems For large-scale problems, sensitive to the number of population
GSA	^86^	Simplicity, flexibility, and ease of implementation Being a memory-less algorithm	Getting easily stuck in local optima Several tuning parameters Slow search speed in final iterations
SCA	^87^	Reasonable time of execution Lower probability of being stuck in local optima Powerful neighborhood exploration characteristics	Suffering from premature convergence Several tuning parameters Probability distribution changes by generations
MOA	^88^	Powerful neighborhood exploration characteristics	Suffering from premature convergence Several tuning parameters Probability distribution changes by generations
ASO	^51^	Appropriate balance between exploration and exploitation abilities Being a memory-less algorithm	Relatively complex Slow convergence rate Several tuning parameters
CryStAl	^52^	Simplicity, flexibility, and ease of implementation Being a parameter-free algorithm Satisfying the local and global searches of the entire search space	Relatively poor performance for some high-dimensional problems Need for a high number of iterations for some examples to find a suitable solution
AEFA	^89^	Simplicity, flexibility, and ease of implementation Good convergence performance in handling some complex optimization problems	Suffering from premature convergence Poor search ability in handling complex optimization problems Several tuning parameters
ICA	^90^	Appropriate convergence rate Strong neighborhood search property	May suffer from premature convergence Several tuning parameters
TLBO	^91^	Being a parameter-free algorithm Appropriate convergence rate Efficient for large-scale problems	Often loses its effectiveness when tackling problems with optima distant from the origin May get stuck in local optima
ISA	^92^	Having only one parameter to be tunned	May get stuck in local optima Suffering from premature convergence
SPO	^60^	Being a parameter-free algorithm Appropriate convergence rate Capability of working with low initial population sizes Simplicity, flexibility, and ease of implementation	May get stuck in local optima for special examples Relatively high computational cost for large-scale problems

The contribution of this paper is to develop a new physics-based metaheuristic algorithm called Fusion Fission Optimization (FuFiO) algorithm. The proposed algorithm simulates the tendency of nuclei to increase their binding energy and achieve higher levels of stability. In the FuFiO algorithm, the nuclei are divided into two groups, namely stable and unstable, based on their fitness. Each nucleus can interact with other nuclei using three different types of nuclear reactions, including fusion, fission, and $\beta$-decay. These reactions establish the stabilization process of unstable nuclei through which they gradually turn into stable nuclei.

The performance of the FuFiO algorithm is also examined and explained in two steps as follows. In the first step, FuFiO and seven other metaheuristic algorithms are used to solve a complete set of 120 benchmark mathematical test functions (including 60 fixed-dimensional and 60 N-dimensional test functions). Then, to make a valid judgment about the performance of the FuFiO algorithm, the obtained statistical results of FuFiO and the other algorithms are utilized as a dataset to be analyzed by non-parametric statistical methods. In the second step, to compare the ability of the proposed algorithm with state-of-the-art algorithms, the single-objective real-parameter numerical optimization problems of the recent Competitions on Evolutionary Computation (CEC 2017) including sets of 10-, 30-, 50-, and 100- dimensional benchmark test functions are considered. It should be noted that in this work, the main novelty is two-fold. First, the source of inspiration is provided by some fundamental aspects of nuclear physics. Second, that is of higher importance and rigor, the theory of nuclear binding energy to generate stable nuclei is used to develop the equations of a metaheuristic method for the first time. In this model, the tendency of nuclei to increase their binding energy and achieve higher levels of stability using nuclear reactions, including fusion, fission, and *β*-decay, is considered the central principle to develop the three main steps of the new algorithm.

The rest of this paper is organized as follows: “Fusion–fission optimization (FuFiO) algorithm” section describes the background, inspiration, mathematical model, and implementation of the proposed algorithm. “FuFiO validation” section explains comparative metaheuristics, mathematical functions, comparative results, and statistical analyses. “Analyses based on competitions on evolutionary computation (CEC)” section compares the performance of the FuFiO algorithm on the CEC-2017 and CEC-2019 special season with state-of-the-art algorithms. Finally, conclusions are given in “Conclusions and future work” section.

## Fusion–fission optimization (FuFiO) algorithm

In the following sub-sections, the general principles of nuclear reactions, nuclear binding energy, and nuclear stability are discussed as an inspirational basis for the development of the Fusion–Fission Optimization (FuFiO) algorithm.

### Inspiration

In nuclear physics, the minimum energy needed to dismantle the *nucleus* of an atom into its constituent *nucleons*, i.e., the collection of *protons* (*Z*) and *neutrons* (*N*), is called *nuclear binding energy*. The strong nuclear force that attracts the nucleons to each other has a positive value and creates this nuclear binding energy. Therefore, a nucleus with more binding energy provides more stability^93^. Importantly, the Coulomb repulsive force of protons reduces the nuclear attraction force and decreases the binding energy. Consequently, the stability of the nucleus further decreases when more protons are replaced with neutrons. Also, in the nuclei, most of the paired protons are close to each other such that their repulsive force decreases the strong nuclear force, leading to instability.

**Figure 1:**
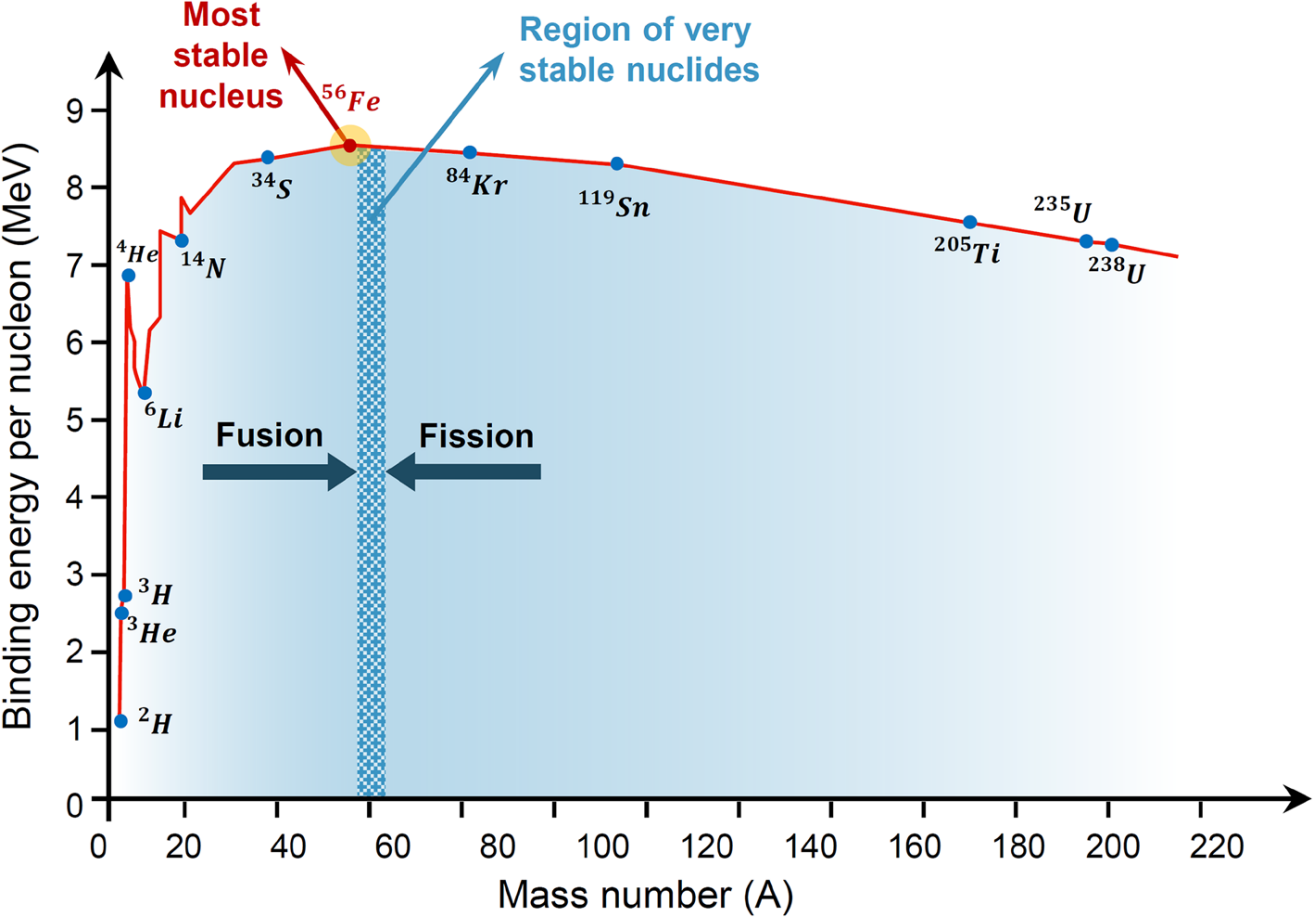
Experimental binding energy ${B}_{Avg}(A, Z)$ with respect to mass number *A*^49^.

The concept of average nuclear binding energy, denoted by ${B}_{Avg}$, is generally used to evaluate the stability of nuclei. ${B}_{Avg}$ is the amount of energy required to disassemble every single nucleon from the nucleus, which is defined as the nuclear binding energy per nucleon in the nucleus. As ${B}_{Avg}$ increases, disassembling every single nucleon from the nucleus becomes progressively more difficult; in other words, the most stable nucleus corresponds to the highest ${B}_{Avg}$. The experimental diagram of ${B}_{Avg}$ associated with mass number $A$ is shown in Fig. 1. According to this diagram, the binding energy reaches its peak at $A=56$ (${}^{56}\mathrm{Fe}$), and in $A>56$, the rate of energy reduction is low, such that the diagram has a relatively flat behavior due to saturation. The ${}^{56}\mathrm{Fe}$ nucleus divides the diagram into two parts, namely fusion and fission. The nuclei of the fusion part tend to participate in a fusion reaction, whereas in the fission part, each nucleus tends to participate in a fission reaction.

**Figure 2:**
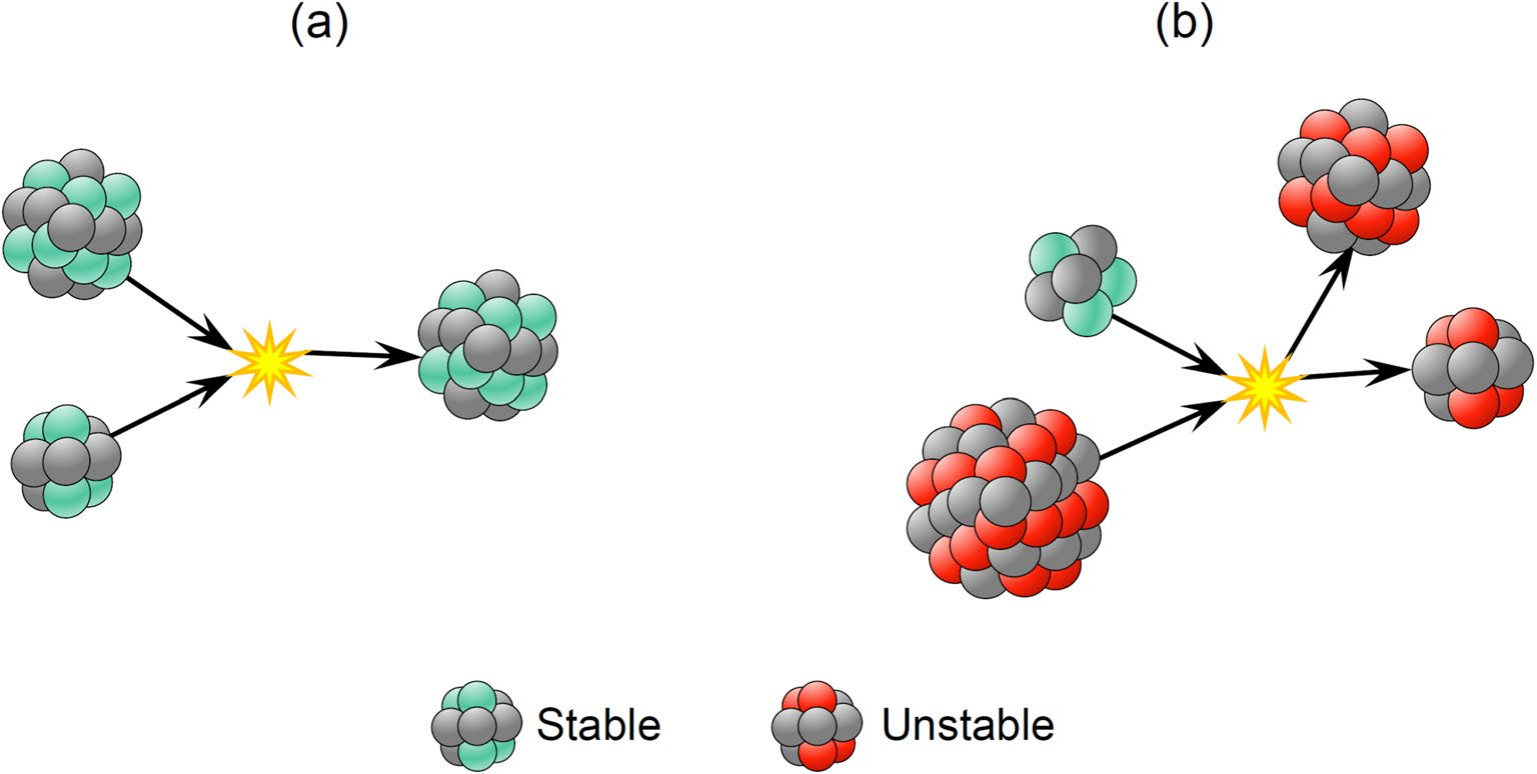
Nuclear reactions: (**a**) fusion, and (**b**) fission.

Fusion is a nuclear reaction and occurs when two highly-energetic stable nuclei slam together to form a heavier stable nucleus. In the sun, this reaction creates a lot of energy through the fusion of two hydrogen nuclei to form one helium nucleus. On the other hand, fission is a nuclear reaction in which a larger unstable nucleus is split into two smaller (stable or unstable) nuclei due to a hit by a smaller stable or unstable one. This type of reaction is used to produce a lot of energy in nuclear power reactors through the fission of Uranium and Plutonium nuclei by neutrons. The procedures of nuclear fusion and fission are illustrated in Fig. 2a,b, respectively.

**Figure 3:**
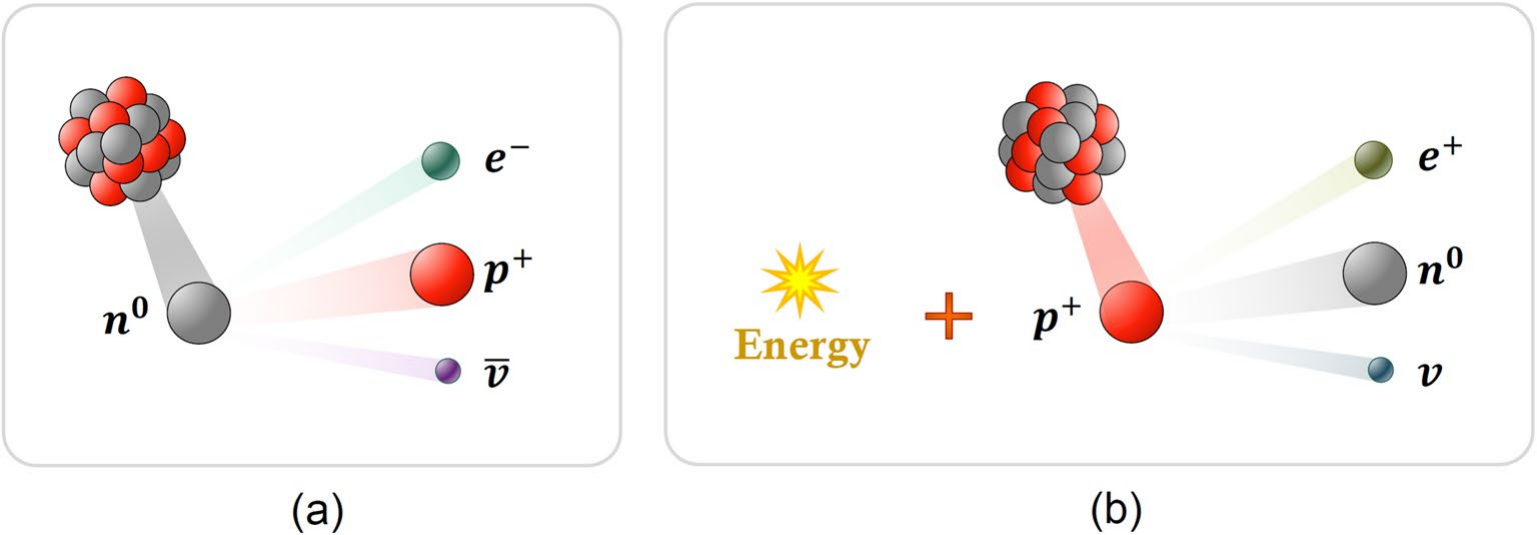
Processes of $\beta$-decay: (**a**) ${\beta }^{-}$-decays, and (**b**) ${\beta }^{+}$-decays.

In nuclear processes, in addition to fusion and fission, there is another process called $\beta$-decay. The two types of $\beta$-decay are known as ${\beta }^{-}$ and ${\beta }^{+}$. In ${\beta }^{-}$-decay, a neutron is converted to a proton, and the process creates an electron and an electron antineutrino ($\overline{v }$), while in ${\beta }^{-}$-decay, a proton is converted to a neutron and the process creates a positron and an electron neutrino ($v$)^94^. Also, neutrino and antineutrino particles have no essential role in reactions because they have considerably smaller masses compared to other particles. Therefore, protons and neutrons are the main factors in ${\beta }^{\pm }$-decays. In Fig. 3, the schematic representations of ${\beta }^{-}$- and ${\beta }^{+}$-decays are presented.

### Mathematical model

**Figure 4:**
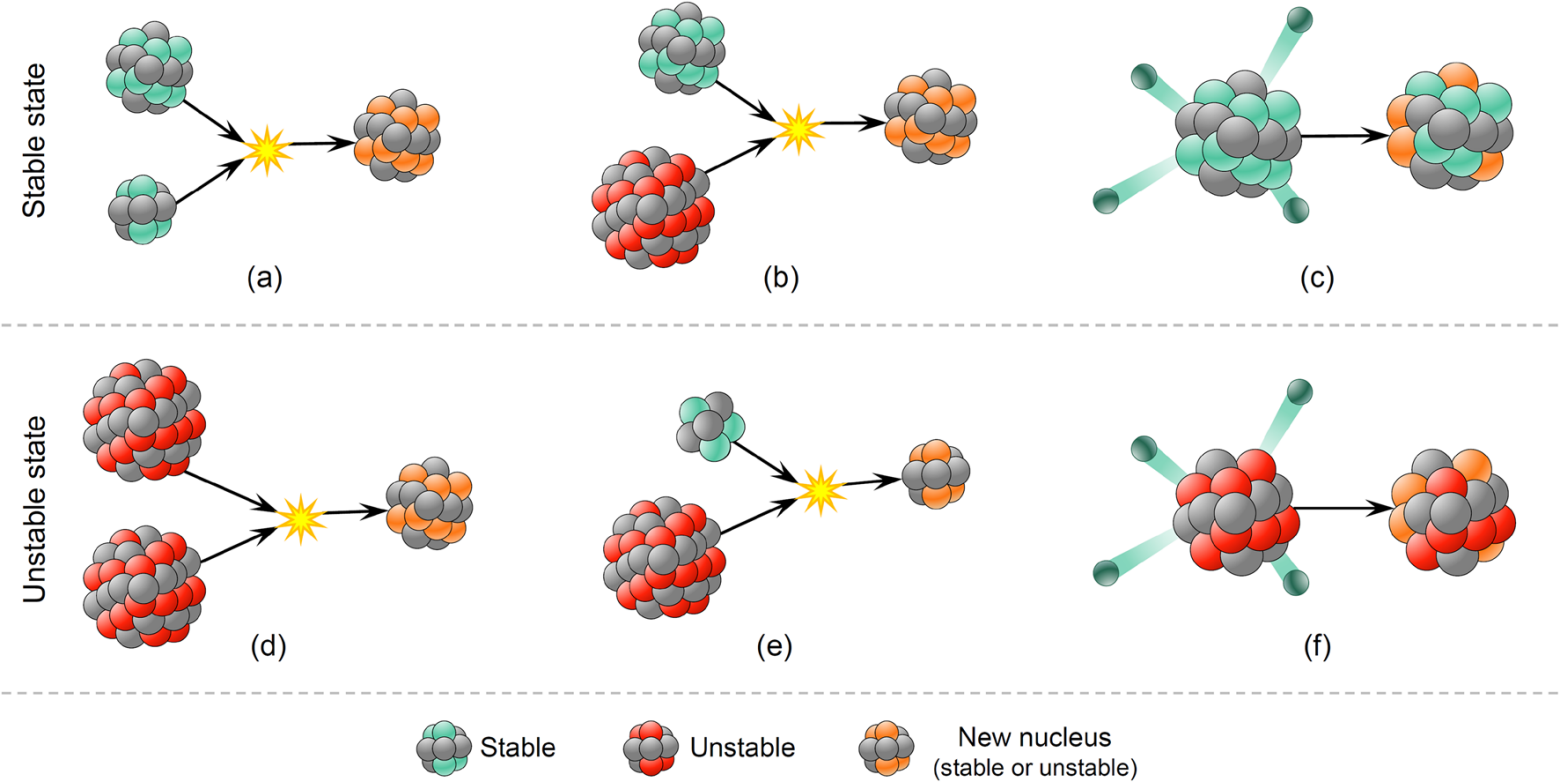
Graphical representation of different reactions in each group of nuclei.

In this section, we describe the mathematical model of the FuFiO algorithm, which is developed based on the tendency of nuclei to increase their binding energy and get a higher level of stability using nuclear reactions, including fusion, fission, and $\beta$-decay. Importantly, as a nucleus with a higher level of binding energy is considered a better solution, the FuFiO algorithm will move in a direction that increases the binding energy of the nuclei. FuFiO is designed as a population-based metaheuristic method in which a set of nuclei are considered as the agents of the population. Each agent of the population has a specific position, and each of them has a particular dimension (*d*) which is determined by the number of problem variables. Therefore, the nuclei move in a *d*-dimensional space, and are represented in the form of a matrix as follows:

1 $$X=\left[\begin{array}{c}{X}_{1}\\ \vdots \\ {X}_{i}\\ \vdots \\ {X}_{n}\end{array}\right]=\left[\begin{array}{cc}\begin{array}{ccc}{x}_{1}^{1}& {x}_{1}^{2}& \cdots \\ \vdots & \vdots & \cdots \\ {x}_{i}^{1}& {x}_{i}^{2}& \cdots \end{array}& \begin{array}{ccc}{x}_{1}^{j}& \cdots & {x}_{1}^{d}\\ \vdots & \cdots & \vdots \\ {x}_{i}^{j}& \cdots & {x}_{i}^{d}\end{array}\\ \begin{array}{ccc}\vdots & \vdots & \cdots \\ {x}_{n}^{1}& {x}_{n}^{2}& \cdots \end{array}& \begin{array}{ccc}\vdots & \cdots & \vdots \\ {x}_{n}^{j}& \cdots & {x}_{n}^{d}\end{array}\end{array}\right]$$

where $i (i = 1, 2, 3, \dots , n)$ is the counter of nucleus and $j(j=1, 2, 3, \dots , d)$ is the counter of design variables; $n$ is the population size; $X$ is the matrix of positions of all nuclei updated in each iteration of algorithm; ${X}_{i}$ is the position of the *i*-th nucleus; and ${x}_{i}^{j}$ is the *j*-th design variable of the *i*-th nucleus the initial value of which is determined randomly as follows:

2 $${x}_{i}^{j}\left(0\right)={lb}^{j}+r({ub}^{j}- {lb}^{j})$$

where ${x}_{i}^{j}\left(0\right)$ represents the initial position of the *j*-th design variable of the *i*-th nucleus; ${ub}^{j}$ and ${lb}^{j}$ are respectively the maximum and minimum possible values for the *j*-th design variable; and $r$ is a random number in the interval [0,1]. The set of initial ${x}_{i}^{j}\left(0\right)$ s will create ${X}^{0}$ that represents the initial position of nuclei. Furthermore, in the FuFiO method, the nuclei are divided into two groups, namely *stable* and *unstable* nuclei, based on the level of binding energy. Depending on the types of reacting nuclei, nuclear reactions (i.e., fusion, fission, and $\beta$-decay) are regarded differently. In other words, as illustrated in Fig. 4, three different types of reaction can be considered in each group for nuclei to update their positions.

The mathematical formulation of each reaction in each group modeled as follows:

**Group 1: Stable nucleus**

**Figure 5:**
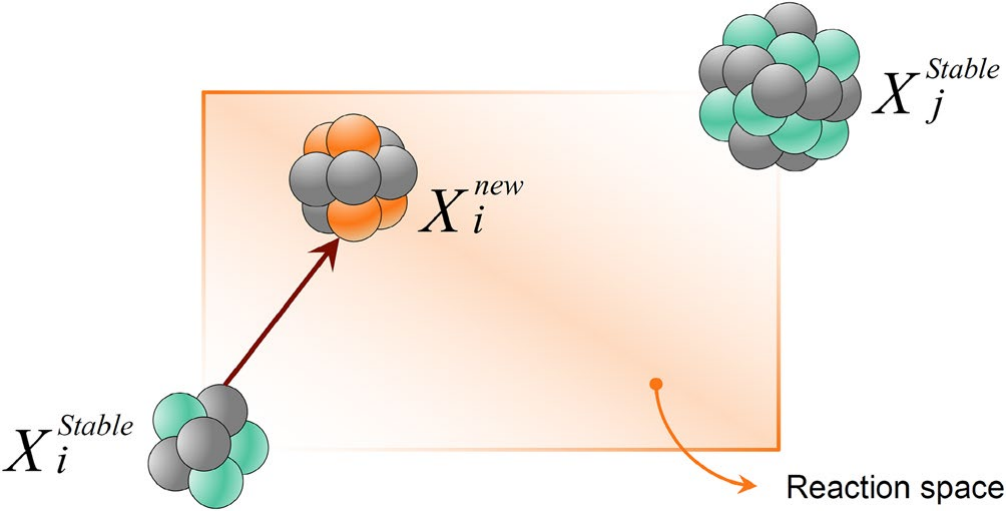
Schematic representation of a fission reaction.

If the *i*-th nucleus is stable (${X}_{i}^{stable}$), one of the following three reactions is selected randomly:

***Reaction 1***: In this reaction, the *i*-th nucleus slams with another stable nucleus. The new position is determined as follows:

3 $${X}_{i}^{new}=r{X}_{i}^{stable}+\left(1-r\right){X}_{j}^{stable}$$

where *r* is a random vector in [0,1] and ${X}_{j}^{stable}$ is a stable nucleus selected randomly from other stable nuclei. This reaction simulates fusion, where two stable nuclei slam together to produce a new nucleus. Figure 5 shows a schematic view of this reaction, from which it can be seen that the new solution is a random point generated in the reaction space using $r$ and $1-r$.

**Figure 6:**
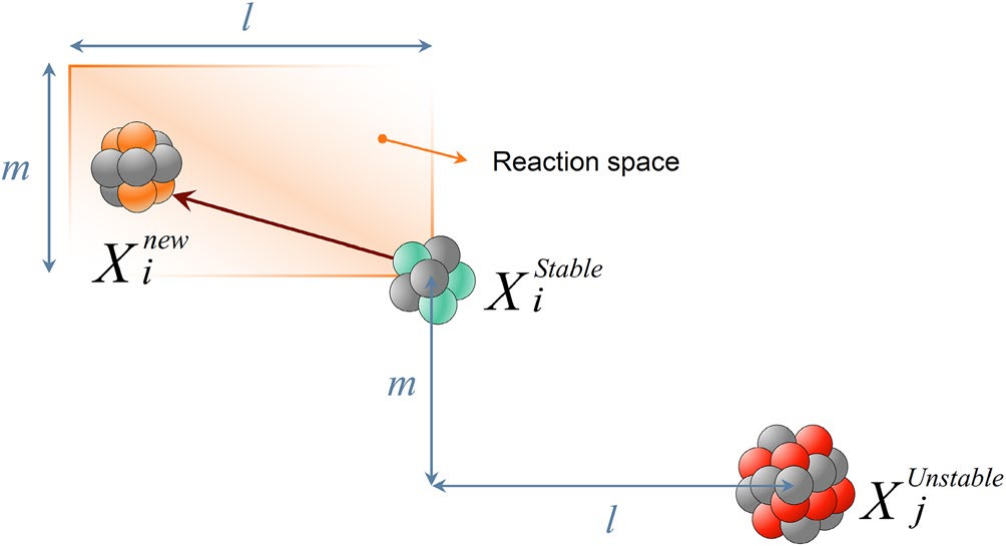
Schematic representation of a fission reaction.

***Reaction 2***: If the *i*-th nucleus interacts with an unstable nucleus, this collision produces a new solution expressed as:

4 $${X}_{i}^{new}={X}_{i}^{stable}+r\left({X}_{i}^{stable}-{X}_{j}^{unstable}\right)$$

where ${X}_{j}^{unstable}$ is an unstable nucleus selected randomly from other unstable nuclei. The process of this reaction, shown in Fig. 6, simulates the rule of fission, where a stable nucleus is hit by an unstable one.

**Figure 7:**
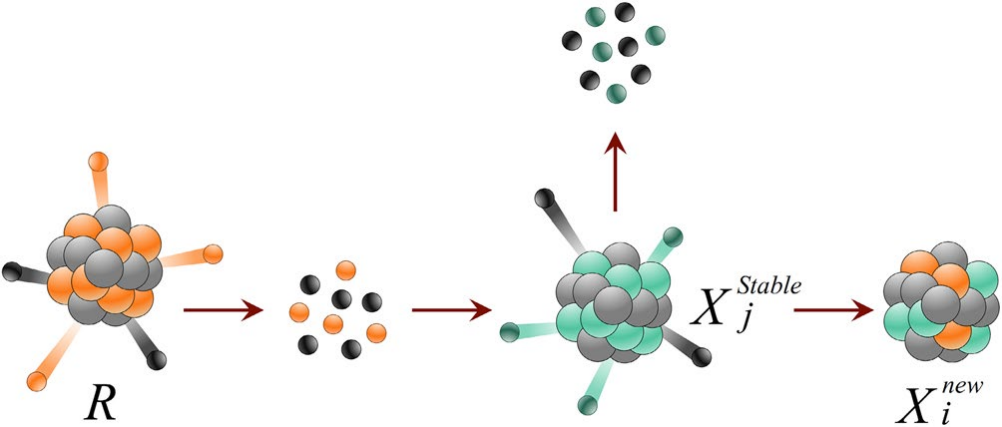
Procedure of $\beta$-decay in a stable nucleus.

***Reaction 3***: If the *i*-th nucleus decays, the new solution will be generated as follows:

$${X}_{i new}^{k}=\left\{\begin{array}{c}{X}_{i}^{k} k\notin p\\ {R}^{k} k\in p\end{array} , p\subseteq d\right.$$

5 $$R=LB+r(UB-LB)$$

where $p$ denotes a random subset of problem variables; $d$ is the set of all variables; $k$ is the counter of variables; $R$ is a random nucleus; and $UB$ and $LB$ are the vectors of the lower and upper bound of variables, respectively. This reaction models the process of $\beta$-decay in a stable nucleus as presented in Fig. 7.

**Group 2: Unstable nucleus**

**Figure 8:**
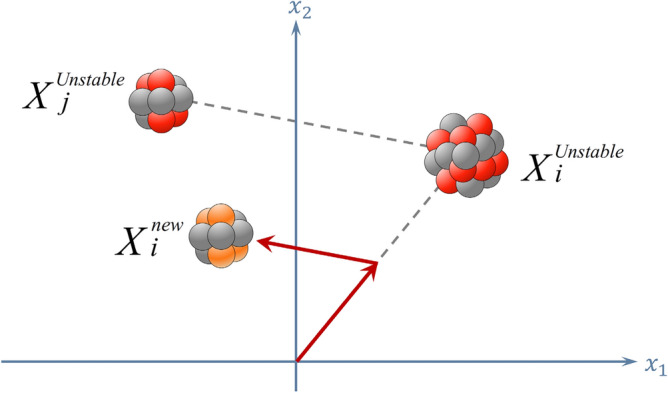
Fission of two unstable nuclei.

In the second group, if the *i*-th nucleus is unstable (${X}_{i}^{unstable}$), one of the following three reactions will be used randomly to update the *i*-th nucleus:

***Reaction 1***: If the unstable nucleus slams with another unstable nucleus, the new position is obtained as follows:

6 $${X}_{i}^{new}=r{X}_{i}^{unstable}+(1-r)({X}_{j}^{unstable}-{X}_{i}^{unstable})$$

where $r$ is a random vector in interval [0,1] and ${X}_{j}^{unstable}$ is an unstable nucleus selected randomly from other unstable nuclei. As illustrated in Fig. 8, this reaction simulates the rule of fission where an unstable nucleus is hit by an unstable one.

**Figure 9:**
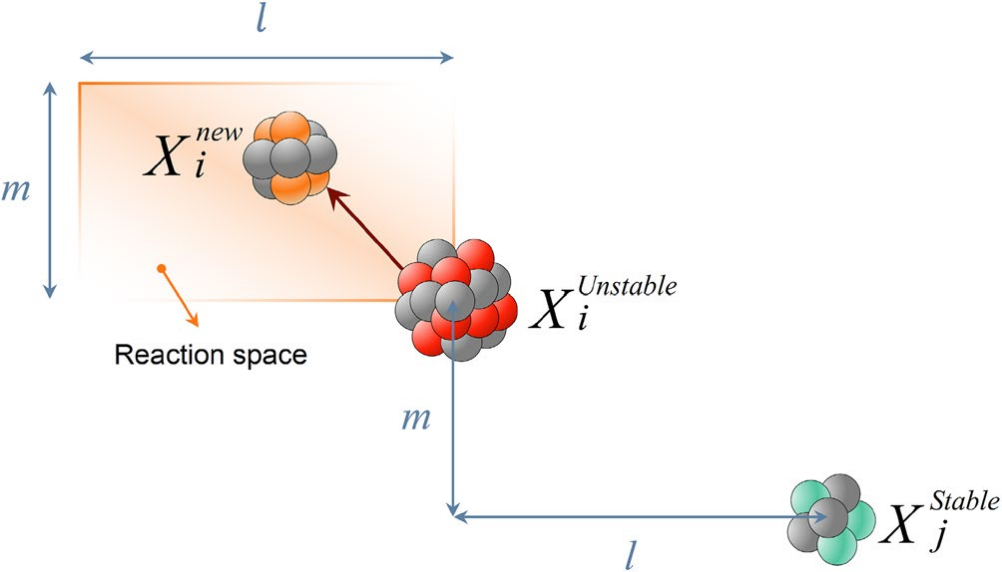
Fission of stable and unstable nuclei.

***Reaction 2***: If the unstable nucleus, ${X}_{i}^{unstable}$, interacts with a stable nucleus, the new position is as follows:

7 $${X}_{i}^{new}={X}_{i}^{unstable}+r({X}_{i}^{unstable}-{X}_{j}^{stable})$$

where ${X}_{j}^{stable}$ is a randomly selected stable nucleus from stable nuclei. The process of this reaction, which establishes a fission model of stable and unstable nuclei, is shown in Fig. 9.

**Figure 10:**
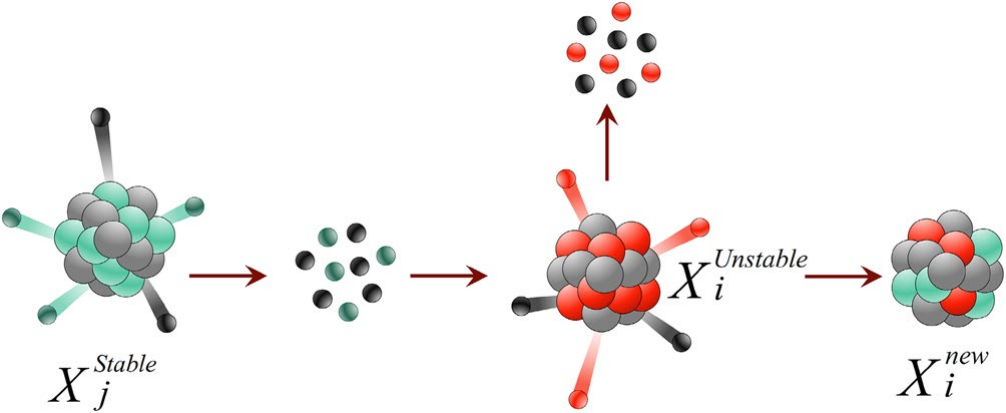
Procedure of *β*-decay in an unstable nucleus.

***Reaction 3***: If the *i*-th unstable nucleus decays, the new position is defined as follows:

8 $${X}_{i new}^{k}=\left\{\begin{array}{l}{X}_{i}^{k}\quad k\notin p\\ {X}_{j}^{k} \quad k\in p\end{array} , p\subseteq d\right.$$

where $p$ denotes a random subset of variables; $d$ is the set of all variables; $k$ is the counter of variables; and ${X}_{j}^{stable}$ is a randomly selected nucleus from stable nuclei. As presented in Fig. 10, this reaction models the $\beta$-decay process of an unstable nucleus.

Both third reactions in the stable and unstable groups represent the ${\beta }^{\pm }$-decays. In the former reaction, a random set of decision variables takes new random values between their corresponding allowable lower and upper bounds, whereas, in the latter one, a random subset of decision variables takes their new values from the corresponding decision variables of a randomly-chosen stable solution. Importantly, the ${\beta }^{\pm }$-decays are considered as *mutation operators* to escape from local optima.

### Stable and unstable nuclei

The level of binding energy of a nucleus determines whether it is stable or unstable, and in the FuFiO algorithm, the objective function value, $F(X)$, is used to specify the group of agents. In other words, in the FuFiO algorithm, a nucleus with a better $F(X)$ is considered to be more stable. Moreover, as can be seen from Fig. 1, the ${}^{56}Fe$ nucleus is the boundary of stable and unstable groups. This boundary is also considered in the FuFiO algorithm to distinguish stable nucleus from unstable ones. To this end, the nucleus is evaluated in each iteration and a set of better ones is considered as the set of stable nuclei. The size of stable nuclei is determined as follows:

9 $${S}_{z}=fix\left[n\times \left({L}_{s}+\frac{Iter\times \left({U}_{s}-{L}_{s}\right)}{MaxIter}\right)\right]$$

where ${S}_{z}$ is the size of stable nuclei at each iteration; $fix$ is a function that rounds its argument to the nearest integer number; $n$ is the population size; ${L}_{s}$ and ${U}_{s}$ are the minimum and maximum percent of stable nuclei at the start and the end of the algorithm, respectively; $Iter$ is the counter of iterations; and $MaxIter$ is the maximum iteration of the algorithm. In Eq. (9), the size of stable particles is determined dynamically as the algorithm progresses. Also, in determining ${S}_{z}$, the two parameters ${L}_{s}$ and ${U}_{s}$ should be fine-tuned. The values of ${L}_{s}$ and ${U}_{s}$ are considered 10% and 70%, respectively. This formulation increases the size of stable nuclei from 10 to 70% at the end of the algorithm. In addition, the value of ${U}_{s}$ is naturally adopted in which the ratio of stable nuclei to unstable nuclei is assumed to be around 70%.

### Boundary handling

In solving an optimization problem with $d$ variables, optimizers search in a *d*-dimensional search space. Each of these dimensions has its upper and lower boundaries, and the variables of found solutions should be placed in the interval of boundaries. Given that some variables may violate boundaries during their movements, in the FuFiO algorithm, the following equations, which replace violated boundaries with violated variables, are used to return them within the boundaries:

10 $${x}_{i new}^{j}=\mathit{min}\left({x}_{i}^{j}, {ub}^{j}\right)\, \mathrm{and}\, {x}_{i new}^{j}=max({x}_{i}^{j}, {lb}^{j})$$

where ${x}_{i new}^{j}$ is the *j*-th design variable of the *i*-th new solution ${X}_{i}^{new}$, and *min* and *max* are operators that return the minimum and maximum of ${(x}_{i}^{j}, {ub}^{j})$ and ${(x}_{i}^{j}, {lb}^{j})$, respectively.

### Replacement strategy

In each reaction, a new position ${X}_{i}^{new}$ is generated to be replaced with the current position of the *i*-th nucleus ${X}_{i}$. This replacement will take place whenever the new solution has a better level of binding energy than the current one. This procedure is formulated as follows:

11 $${X}_{i}=\left\{\begin{array}{c}{X}_{i} {\quad}{\quad} f\left({X}_{i}\right)\, is \, better \, than \, f\left({X}_{i}^{new}\right)\\ {X}_{i}^{new} {\quad}{\quad}f\left({X}_{i \, new}\right)\, is \, better \, than \, f\left({X}_{i}\right)\end{array}\right.$$

### Selection of reactions

**Figure 11:**
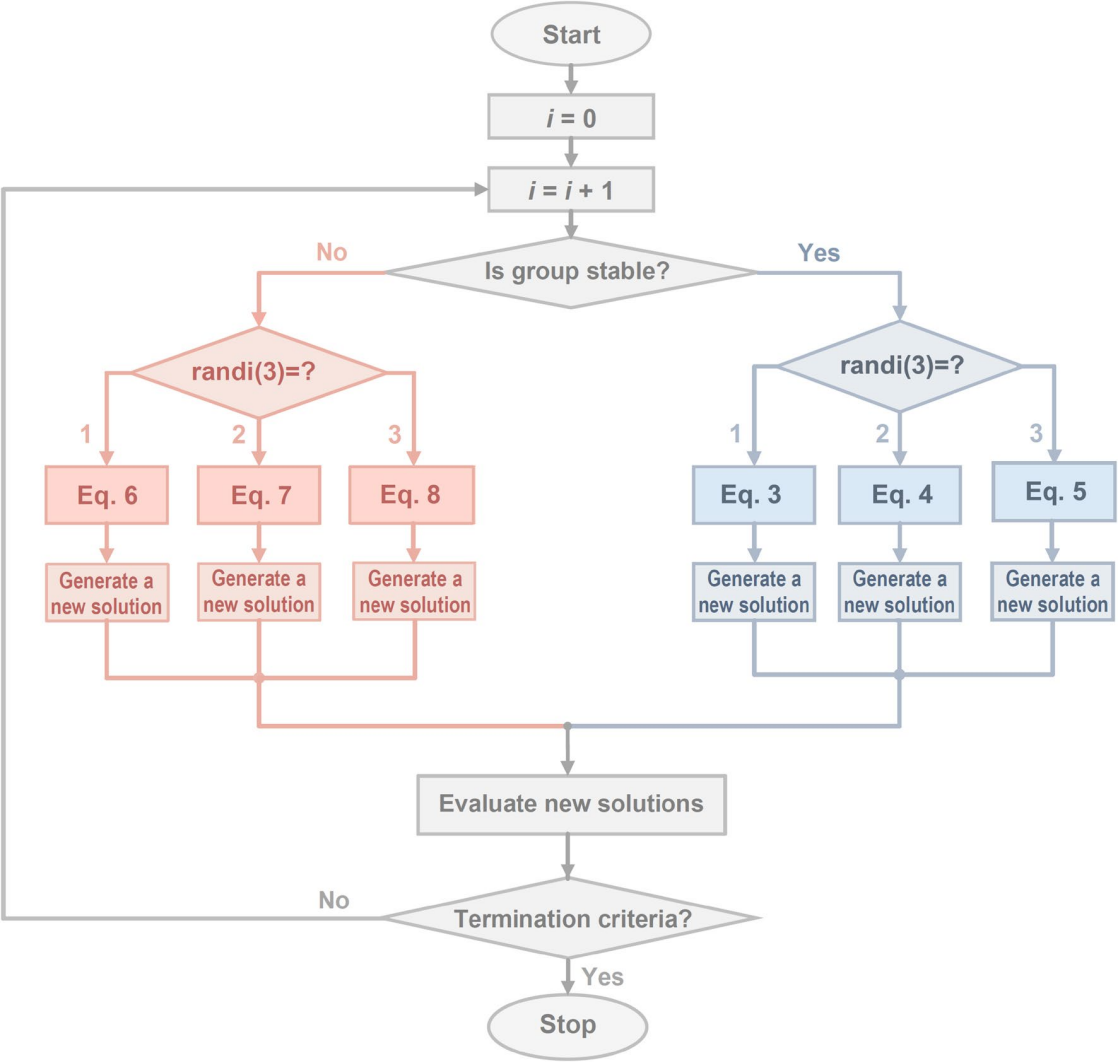
Flowchart of the process of determining groups and reactions in each iteration for each agent.

In the FuFiO algorithm, nuclei are categorized into two groups; in each group, three different reactions are developed, of which one is randomly selected to generate a new solution. It should be noted that different groups and reactions do not represent different phases of the algorithm. In other words, the FuFiO algorithm has one phase, wherein for each nucleus in each iteration, one of the reactions is randomly selected according to the group of the nucleus to generate the new solutions, as shown in Fig. 11.

### Terminating criterion

In metaheuristics, the search process will be finished after satisfying a terminating criterion, following that the best result will be reported. Some of the most common stop criteria are as follows:

- The best result is equal to the minimum specified value determined for the objective function.
- The optimization process will be terminated after a fixed number of iterations.
- The value of the objective function does not change during the specified period.
- The optimization process time has reached a predetermined value.

### Implementation of FuFiO

Based on the concepts developed in previous sections, the FuFiO algorithm is implemented in two levels as follows:

**Level 1: Initialization**

- **Step 1**: Determine the number of nucleus ($nPop$), maximum number of iterations ($MaxIter$), and variable bounds $UB$ and $LB$.
- **Step 2**: Determine the parameters of FuFiO, namely ${L}_{s}$ and ${U}_{s}$.
- **Step 3**: Define initial solutions (Eqs. (1) and (2)).
- **Step 4**: Calculate the objective function of initial solutions.

**Level 2: Nuclear reaction**

In each iteration of the FuFiO algorithm, all of the agents will perform the following steps:

- **Step 1**: ${S}_{z}$ is updated (Eq. (9)).
- **Step 2**: Population is sorted according to $F(X)$.
- **Step 3**: Stable and unstable nuclei are determined.
- **Step 4**: The group of current nucleus is determined.
- **Step 5**: The new solution is generated using the selected reaction (Eqs. (3), (4), (5), (6), (7), and (8)).
- **Step 6**: The new solution is clamped as Eq. (10).
- **Step 7**: The new solution is evaluated and objective function $F(X)$ is calculated.
- **Step 8**: The new solution is checked to replace the current solution as Eq. (11).
- **Step 9**: Nuclear reaction level is repeated until a terminating criterion is satisfied.

**Figure 12:**
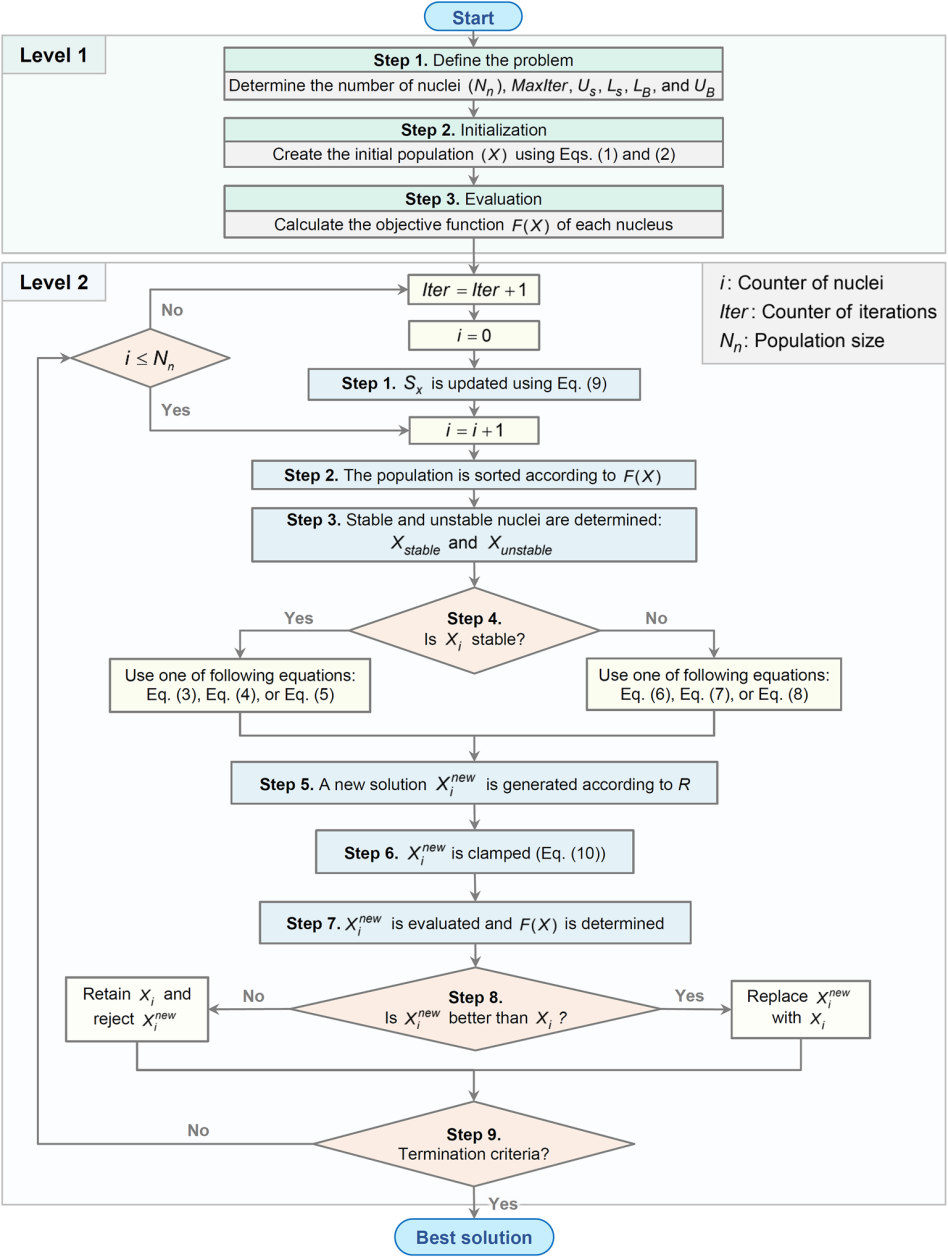
Flowchart of the Fusion–Fission Optimization (FuFiO) algorithm.

The flowchart of the FuFiO algorithm is illustrated in Fig. 12.

## FuFiO validation

The No Free Lunch (NFL) theorem^95^ is one of the most famous theories which have been cited many times in literature to pave the way for introducing new metaheuristic algorithms. This theorem has logically proved that no algorithm can solve all types of problems. However, the NFL theorem is used here for a different purpose. In other words, it is used here to validate the capability of the FuFiO algorithm in solving various problems compared to other algorithms. To this end, in this study, 120 benchmark test functions are considered to challenge the performance of the proposed algorithm in solving different types of problems. Also, another application of these problems is to create a dataset to be used in non-parametric statistical analyses to examine the performance of the proposed algorithm more thoroughly.

In this section, first, the description of the test problems is presented; then, a number of rival metaheuristics with their settings are reviewed. Subsequently, the evaluation metrics and comparative results are explained; and finally, the results of non-parametric statistical methods will be presented.

### Test functions

To evaluate the capability of the proposed algorithm in handling various types of benchmark functions with different properties, a set of 120 mathematical problems has been used. Based on their dimensions, these problems have been categorized into two groups: (1) fixed-dimensional problems, and (2) *N*-dimensional problems.

Amongst these functions, *F*_1_ to *F*_60_ are fixed-dimensional functions, with dimensions of 2 to 10. The second group of problems, *F*_61_ to *F*_120_, includes 60 N-dimensional test functions, the dimensions of which are considered to be equal to 30. The details of the mathematical functions in these two groups are presented in Tables 2 and 3, respectively. In these tables, C, NC, D, ND, S, NS, Sc, NSC, U, and M denote Continuous, Non-Continuous, Differentiable, Non-Differentiable, Separable, Non-Separable, Scalable, Non-Scalable, Unimodal, and Multi-modal, respectively. In addition, *R*, *D*, and Min represent the variables range, variables dimension, and the global minimum of the functions, respectively.

**Table 2:** Details of the fixed-dimensional benchmark mathematical functions.

No	Function	Type	Range	*D*	Formulation	Min
F1	Ackley 2 Function	C, D, NS, Sc, M	[− 35, 35]	2	^96^	− 200
F2	Ackley 3 Function	C, D, NS, NSc, U	[− 32, 32]	2	^96^	− 195.629
F3	Ackley 4 or Modified Ackley	C, D, NS, Sc, M	[− 32, 32]	2	^96^	− 4.590102
F4	Adjiman Function	C, D, NS, NSc, M	[− 1, 2] and [− 1, 1]	2	^96^	− 2.021807
F5	Bartels Conn Function	C, ND, NS, NSc, M	[− 500, 500]	2	^96^	1
F6	Bohachevsky 1 Function	C, D, S, NSc, M	[− 100, 100]	2	^96^	0
F7	Bohachevsky 2 Function	C, D, NS, NSc, M	[− 100, 100]	2	^96^	0
F8	Bohachevsky 3 Function	C, D, NS, NSc, M	[− 100, 100]	2	^96^	0
F9	Camel Function-Three Hump	C, D, NS, NSc, M	[− 5, 5]	2	^96^	0
F10	Carrom table function	NS	[− 10, 10]	2	^96^	− 24.15682
F11	Chichinadze Function	C, D, S, NSc, M	[− 30, 30]	2	^96^	− 43.72192
F12	Cross-in-Tray Function	C, NS, NSc, M	[− 10, 10]	2	^96^	− 2.062612
F13	Cube Function	C, D, NS, NSc, U	[− 10, 10]	2	^96^	0
F14	Damavandi Function	C, D, NS, NSc, M	[0, 14]	2	^96^	0
F15	Deckkers–Aarts Function	C, D, NS, NSc, M	[− 20, 20]	2	^96^	− 24,776.52
F16	Egg Crate Function	C, D, NS, Sc, M	[− 5, 5]	2	^96^	0
F17	Giunta Function	C, D, S, Sc, M	[− 1, 1]	2	^96^	0.0644704
F18	Hansen Function	C, D, S, NSc, M	[− 10, 10]	2	^96^	− 166.0291
F19	Himmelblau Function	C, D, NS, NSc, M	[− 5, 5]	2	^96^	0
F20	Hosaki Function	C, D, NS, NSc, M	[0, 5] and [0, 6]	2	^96^	− 2.3458
F21	Jennrich–Sampson Function	C, D, NS, NSc, M	[− 1, 1]	2	^96^	124.36218
F22	Keane Function	C, D, NS, NSc, M	[0, 10]	2	^96^	− 0.673668
F23	Leon Function	C, D, NS, NSc, U	[− 1.2, 1.2]	2	^96^	0
F24	Levy 3 Function	S	[− 10, 10]	2	^97^	− 176.5418
F25	Levy 5 Function	NS	[− 10, 10]	2	^97^	− 176.1376
F26	Matyas Function	C, D, NS, NSc, U	[− 10, 10]	2	^96^	0
F27	McCormick Function	C, D, NS, NSc, M	[− 1.5, 4] and [− 3, 3]	2	^96^	− 1.913223
F28	Mexican hat Function	NS	[− 10, 10]	2	^97^	− 19.96668
F29	Michaelewicz 2 Function	S	[0, π]	2	^97^	− 1.8013
F30	Mishra 5 Function	C, D, NS, NSc, M	[− 10, 10]	2	^96^	− 1.01983
F31	Mishra 6 Function	C, D, NS, NSc, M	[− 10, 10]	2	^96^	− 2.28395
F32	Mishra 8 Function	C, D, NS, NSc, M	[− 10, 10]	2	^96^	0
F33	Pen Holder Function	C, D, NS, NSc, M	[− 11, 11]	2	^96^	− 0.963535
F34	Periodic Function	S	[− 10, 10]	2	^97^	0.9
F35	Price 1 Function	C, ND, S, NSc, M	[− 500, 500]	2	^96^	0
F36	Price 2 Function	C, D, NS, NSc, M	[− 10, 10]	2	^96^	0.9
F37	Price 4 Function	C, D, NS, NSc, M	[− 500, 500]	2	^96^	0
F38	Quadratic Function	C, D, NS, NSc	[− 10, 10]	2	^96^	− 3873.724
F39	Ripple 1 Function	NS	[0, 1]	2	^97^	− 2.2
F40	Ripple 25 Function	NS	[0, 1]	2	^97^	− 2
F41	Rosenbrock Modified Function	C, D, NS, NSc, M	[− 2, 2]	2	^96^	34.040243
F42	Rotated Ellipse Function	C, D, NS, NSc, U	[− 500, 500]	2	^96^	0
F43	Rotated Ellipse 2 Function	C, D, NS, NSc, U	[− 500, 500]	2	^96^	0
F44	Scahffer 2 Function	C, D, NS, NSc, U	[− 100, 100]	2	^96^	0
F45	Scahffer 3 Function	C, D, NS, NSc, U	[− 100, 100]	2	^96^	0.0015669
F46	Scahffer 4 Function	C, D, NS, NSc, U	[− 100, 100]	2	^96^	0.292579
F47	Table 1/Holder Table 1 Function	C, D, S, NSc, M	[− 10, 10]	2	^96^	− 26.92034
F48	Table 2/Holder Table 2 Function	C, D, S, NSc, M	[− 10, 10]	2	^96^	− 19.2085
F49	Table 3/Carrom Table Function	C, D, NS, NSc, M	[− 10, 10]	2	^96^	− 24.15682
F50	Ursem 1 Function	S	[− 2.5, 3] and [− 2, 2]	2	^97^	− 4.816814
F51	Ursem 3 Function	NS	[− 2, 2] and [− 1.5, 1.5]	2	^97^	− 3
F96	Ursem 4 Function	NS	[− 2, 2]	2	^97^	− 1.5
F53	Ursem Waves Function	NS	[− 0.9, 1.2] and [− 1.2, 1.2]	2	^97^	− 8.5536
F54	Venter Sobiezcczanski-Sobieski Function	C, D, S, NSc	[− 50, 50]	2	^96^	− 400
F55	Wayburn Seader 3 Function	C, D, NS, Sc, U	[− 500, 500]	2	^96^	19.10588
F56	Zettl Function	C, D, NS, NSc, U	[− 5, 10]	2	^96^	− 0.003791
F57	Zirilli or Aluffi-Pentini’s Function	C, D, S, NSc, U	[− 10, 10]	2	^96^	− 0.352386
F58	Zirilli Function 2	C, D, S, S, M	[− 500, 500]	2	^96^	0
F59	Corana Function	DC, ND, S, Sc, M	[− 500, 500]	4	^96^	0
F60	Michalewicz 10	S	[0, $\pi$]	10	^97^	− 9.66015

**Table 3:** Details of the *N*-dimensional benchmark mathematical functions.

No	Function	Type	Range	*D*	Formulation	Min
F61	Ackley 1 Function	C, D, NS, Sc,M	[− 35, 35]	30	^96^	0
F62	Alpine 1 Function	C, ND, S, NSc,U	[− 10, 10]	30	^96^	0
F63	Brown Function	C, D, NS, Sc, U	[− 1, 4]	30	^96^	0
F64	Chung Reynolds Function	C, D, PS, Sc, U	[− 100, 100]	30	^96^	0
F65	Cosine Mixture	C, ND, S, Sc, M	[− 1, 1]	30	^96^	− 3
F66	Csendes Function	C, D, S, Sc, M	[− 1, 1]	30	^96^	0
F67	Deb 1 Function	C, D, S, Sc, M	[− 1, 1]	30	^96^	− 1
F68	Deb 3 Function	C, D, S, Sc, M	[0, 1]	30	^96^	− 1
F69	Dixon and Price Function	C, D, NS, Sc, U	[− 10, 10]	30	^96^	0
F70	Exponential Function	C, D, NS, Sc, M	[− 1, 1]	30	^96^	− 1
F71	Griewank Function	C, D, NS, Sc, M	[− 100,100]	30	^96^	0
F72	Holzman 2 Function	S	[− 10, 10]	30	^97^	0
F73	Levy 8 Function	NS	[− 10, 10]	30	^97^	0
F74	Mishra 1 Function	C, D, NS, Sc, M	[0, 1]	30	^96^	2
F75	Mishra 2 Function	C, D, NS, Sc, M	[0, 1]	30	^96^	2
F76	Mishra 7 Function	C, D, NS, NSc, M	[− 10, 10]	30	^96^	0
F77	Mishra 11 Function	C, D, NS, NSc, M	[− 10, 10]	30	^96^	0
F78	Pathological Function	C, D, NS, NSc, M	[− 100, 100]	30	^96^	0
F79	Pint´er Function	C, D, NS, Sc, M	[− 10, 10]	30	^96^	0
F80	Powell Singular Function	C, D, NS, Sc, U	[− 4, 5]	30	^96^	0
F81	Powell Singular 2 Function	C, D, NS, Sc, U	[− 4, 5]	30	^96^	0
F82	Powell Sum Function	C, D, S, Sc, U	[− 1, 1]	30	^96^	0
F83	Rastrigin Function	C, D, S, M	[− 5.12, 5.12]	30	^96^	0
F84	Qing Function	C, D, S, Sc, M	[− 500, 500]	30	^96^	0
F85	Quartic	C, D, S, Sc	[− 1.28, 1.28]	30	^96^	0
F86	Quintic Function	C, D, S, NSc, M	[− 10, 10]	30	^96^	0
F87	Rosenbrock Function	C, D, NS, Sc, U	[− 30, 30]	30	^96^	0
F88	Salomon Function	C, D, NS, Sc, M	[− 100, 100]	30	^96^	0
F89	Sargan	C, D, NS, Sc, M	[− 100, 100]	30	^96^	0
F90	Schumer Steiglitz Function	C, D, S, Sc, U	[− 100, 100]	30	^96^	0
F91	Schwefel Function	C, D, PS, Sc, U	[− 100, 100]	30	^96^	0
F92	Schwefel 1.2 Function	C, D, NS, Sc, U	[− 100, 100]	30	^96^	0
F93	Schwefel 2.4 Function	C, D, S, NSc, M	[0, 10]	30	^96^	0
F94	Schwefel 2.20 Function	C, ND, S, Sc, U	[− 100, 100]	30	^96^	0
F95	Schwefel 2.21 Function	C, ND, S, Sc, U	[− 100, 100]	30	^96^	0
F96	Schwefel 2.22 Function	C, D, NS, Sc, U	[− 100, 100]	30	^96^	0
F97	Schwefel 2.23 Function	C, D, NS, Sc, U	[− 10, 10]	30	^96^	0
F98	Schwefel 2.26 Function	C, D, S, Sc, M	[− 500, 500]	30	^96^	− 418.9828
F99	Shubert	C, D, S, NSc, M	[− 10, 10]	30	^96^	− 186.7309
F100	Shubert 3	C, D, S, NSc, M	[− 10, 10]	30	^96^	− 29.6759
F101	Shubert 4	C, D, S, NSc, M	[− 10, 10]	30	^96^	− 25.74177
F102	Schaffer F6	C, D, NS, Sc, M	[− 100, 100]	30	^96^	0
F103	Sphere Function	C, D, S, Sc, M	[0, 10]	30	^96^	0
F104	Step Function	DC, ND, S, Sc, U	[− 100, 100]	30	^96^	0
F105	Step 2 Function	DC, ND, S, Sc, U	[− 100, 100]	30	^96^	0
F106	Step 3 Function	DC, ND, S, Sc, U	[− 100, 100]	30	^96^	0
F107	Stepint Function	DC, ND, S, Sc, U	[− 5.12, 5.12]	30	^96^	− 155
F108	Streched V Sine Wave Function	C, D, NS, Sc, U	[− 10, 10]	30	^96^	0
F109	Sum Squares Function	C, D, S, Sc, U	[− 10, 10]	30	^96^	0
F110	Styblinski–Tang Function	C, D, NS, NSc, M	[− 5, 5]	30	^96^	− 1174.985
F111	Trigonometric 1 Function	C, D, NS, Sc, M	[0, π]	30	^96^	0
F112	Trigonometric 2 Function	C, D, NS, Sc, M	[− 500, 500]	30	^96^	1
F113	W/Wavy Function	C, D, S, Sc, M	[− π, π]	30	^96^	0
F114	Weierstrass	C, D, S, Sc, M	[− 0.5, 0.5]	30	^96^	0
F115	Whitley	C, D, NS, Sc, M	[− 10.24, 10.24]	30	^96^	0
F116	Xin-She Yang (Function 1)	DC, ND, NS, Sc, M	[− 20, 20]	30	^96^	0
F117	Xin-She Yang (Function 2)	DC, ND, NS, Sc, M	[− 10, 10]	30	^96^	0
F118	Xin-She Yang (Function 3)	DC, ND, NS, Sc, M	[− 2π, 2π]	30	^96^	− 1
F119	Xin-She Yang (Function 4)	DC, ND, NS, Sc, M	[− 5, 5]	30	^96^	− 1
F120	Zakharov Function	C, D, NS, Sc, M	[− 5, 10]	30	^96^	0

### Metaheuristic algorithms for comparative studies

To investigate the overall performance of the FuFiO algorithm, its results should be compared with those of other methods. The selected metaheuristics for this purpose are FA, CS, Jaya, TEO, SCA, MVO, and CSA algorithms, of which the most recent and improved versions are utilized here. Among the selected methods, only SCA is parameter-free, whereas the other metaheuristics have some specific parameters that should be tuned carefully. Table 4 presents a summary of these parameters, adopted from the literature, that we have utilized in our evaluations.

**Table 4:** Summary of parameters associated with the methods used for comparative analyses.

Metaheuristic	Parameters	Description	Value
FA	$\gamma$	Light absorption coefficient	1
	$\beta$	Attraction coefficient base value	2
	$\alpha$	Mutation coefficient	0.2
	${\alpha }_{damp}$	Mutation coefficient damping ratio	0.98
	$\delta$	Uniform mutation range	0.05
CS	*p*	Discovery rate of alien eggs	0.25
TEO	${c}_{1}$	Controlling parameters	rand
	${c}_{2}$	Controlling parameters	rand
	${S}_{TM}$	Thermal memory size	5
	$Pro$	Mutation probability	0.05
MVO	${WEP}_{max}$	Maximum Wormhole Existence Probability	1.0
	${WEP}_{min}$	Minimum Wormhole Existence Probability	0.2
	$p$	Exploitation accuracy	1/6
CSA	*ap*	Awareness probability	0.10
	*fl*	Flight length	2.00
FuFiO	${U}_{s}$	Maximum percent of stable nuclei	70%
	${L}_{s}$	Minimum percent of stable nuclei	10%

Generally speaking, the performance of a powerful and versatile algorithm should be independent of the problem that is to be solved. In other words, for a good algorithm, parameter tunning should not be of crucial importance. Considering this point, we developed the FuFiO algorithm in a way that there are only two extra parameters, namely *L*_*s*_ and *U*_*s*_. We performed a statistical study on the effect of these parameters and found out that if they are chosen from within predefined limits, determining the exact values of them is not necessary. Knowing that *L*_*s*_ and *U*_*s*_ are respectively the minimum and maximum percentages of stable nuclei at the beginning and end of the algorithm, *L*_*s*_ should be a small value, e.g. 0.1–0.4, whereas *U*_*s*_ should be in the range of 0.5–0.9. In this study, we considered *L*_*s*_ and *U*_*s*_ to be 0.1 and 0.7, respectively.

### Numerical results

This section presents the results of the FuFiO and other methods in dealing with benchmark problems. In this study, due to the random nature of metaheuristics, each algorithm is independently run 50 times for each problem. Then, the statistical results of these runs are utilized to analyze the algorithms. The population size for each of the methods is set to be 50, and the maximum Number of Function Evaluations (NFEs) is considered 150,000 for all of the metaheuristics. The tolerance of 1 × 10^−12^ from the optimal solution is considered as the terminating criterion, and the NFEs are counted until the algorithm stops. The statistical results of the fixed-dimensional and *N*-dimensional benchmark problems are presented in Tables 5 and 6, respectively. These results include the minimum (Min), average (Mean), maximum (Max), Standard deviation (Std. Dev.), and mean of the NFEs of each algorithm. Moreover, the last row of each function shows the rank of algorithms, where the ranking is based on the value of the Means.

**Table 5:** Comparative results of algorithms for the fixed-dimensional functions.

No	Statistics	Methods							
		FA	CS	Jaya	TEO	SCA	MVO	CSA	FuFiO
F1	Min	− 199.99977	− 200	− 200	− 200	− 200	− 199.99997	− 200	− 200
	Mean	− 199.99853	− 200	− 200	− 200	− 200	− 199.99925	− 200	− 200
	Max	− 199.99688	− 200	− 200	− 200	− 200	− 199.99822	− 200	− 200
	Std. Dev	0.0006252	0	0	0	0	0.000408	0	0
	NFEs	150,875.42	55,584	11,994	24,204	12,588	150,000	63,892	2364
	Rank	8	3.5	3.5	3.5	3.5	7	3.5	3.5
F2	Min	− 195.62903	− 195.62903	− 195.62903	− 195.62903	− 195.62903	− 195.62903	− 195.62903	− 195.62903
	Mean	− 195.62903	− 195.62903	− 195.62902	− 195.61823	− 195.629	− 195.62903	− 195.62903	− 195.62903
	Max	− 195.62902	− 195.62903	− 195.62899	− 195.55082	− 195.62893	− 195.62903	− 195.62903	− 195.62903
	Std. Dev	1.039E−06	2.842E−13	1.222E−05	0.0176225	2.404E−05	3.938E−07	2.842E−13	8.527E−14
	NFEs	150,854	28,512	150,000	150,000	149,950	150,000	11,453	127,158
	Rank	5	2.5	6	8	7	4	2.5	1
F3	Min	− 4.5901016	− 4.5901016	− 4.5901016	− 4.5901016	− 4.590101	− 4.5901016	− 4.5901016	− 4.5901016
	Mean	− 4.5901001	− 4.5901016	− 4.590035	− 4.5900936	− 4.5900145	− 4.5901013	− 4.5901016	− 4.5901016
	Max	− 4.5900934	− 4.5901016	− 4.5895858	− 4.5900376	− 4.5898699	− 4.5900999	− 4.5901016	− 4.5901016
	Std. Dev	1.501E−06	6.217E−15	8.846E−05	9.997E−06	6.885E−05	3.267E−07	6.217E−15	6.217E−15
	NFEs	150,841.6	28,450	150,000	150,000	149,950	150,000	10,339	141,602
	Rank	5	2	7	6	8	4	2	2
F4	Min	− 2.0218068	− 2.0218068	− 2.0218068	− 2.0218068	− 2.0218068	− 2.0218068	− 2.0218068	− 2.0218068
	Mean	− 2.0218068	− 2.0218068	− 2.0218068	− 2.0218066	− 2.0218068	− 2.0218068	− 2.0218068	− 2.0218068
	Max	− 2.0218068	− 2.0218068	− 2.0218068	− 2.0218046	− 2.0218068	− 2.0218068	− 2.0218068	− 2.0218068
	Std. Dev	8.882E−16	8.882E−16	8.882E−16	4.395E−07	1.514E−10	3.024E−13	6.809E−12	8.882E−16
	NFEs	36,849.12	8206	1800	150,000	139,121	126,476	148,460	104,465
	Rank	2.5	2.5	2.5	8	7	5	6	2.5
F5	Min	1.0000886	1	1	1	1	1.0000304	1	1
	Mean	1.0007808	1	1	1	1	1.0004954	1	1
	Max	1.0021551	1	1	1	1	1.0021724	1	1
	Std. Dev	0.0004981	0	0	0	0	0.0003655	0	0
	NFEs	150,906.34	42,278	10,143	23,626	10,084	150,000	50,095	1963
	Rank	8	3.5	3.5	3.5	3.5	7	3.5	3.5
F6	Min	4.745E−08	0	0	0	0	1.863E−07	0	0
	Mean	3.164E−05	0	0	0	0	1.021E−05	0	0
	Max	0.0001135	0	0	0	0	3.597E−05	0	0
	Std. Dev	2.842E−05	0	0	0	0	9.096E−06	0	0
	NFEs	150,869.36	28,000	7968	24,170	6469	150,000	13,524	1407
	Rank	8	3.5	3.5	3.5	3.5	7	3.5	3.5
F7	Min	2.619E−07	0	0	0	0	8.751E−08	0	0
	Mean	2.058E−05	0	0	0	0	1.007E−05	0	0
	Max	0.0001665	0	0	0	0	3.131E−05	0	0
	Std. Dev	2.807E−05	0	0	0	0	8.73E−06	0	0
	NFEs	150,879.44	29,616	9088	24,543	7163	150,000	13,637	1442
	Rank	8	3.5	3.5	3.5	3.5	7	3.5	3.5
F8	Min	1.433E−07	0	0	0	0	8.879E−08	0	0
	Mean	8.802E−06	0	0	0	0	4.63E−06	0	0
	Max	3.256E−05	0	0	0	0	1.745E−05	0	0
	Std. Dev	8.204E−06	0	0	0	0	4.059E−06	0	0
	NFEs	150,716.78	28,952	13,880	24,182	8836	150,000	12,687	1732
	Rank	8	3.5	3.5	3.5	3.5	7	3.5	3.5
F9	Min	7.769E−11	0	0	0	0	1.28E−11	0	0
	Mean	4.322E−09	0	0	0	0	1.537E−09	0	0
	Max	2.212E−08	0	0	0	0	5.801E−09	0	0
	Std. Dev	4.395E−09	0	0	0	0	1.312E−09	0	0
	NFEs	150,853.8	20,260	6961	18,743	4363	150,000	7161	1133
	Rank	8	3.5	3.5	3.5	3.5	7	3.5	3.5
F10	Min	− 24.156816	− 24.156816	− 24.156816	− 24.156811	− 24.156495	− 24.156816	− 24.156816	− 24.156816
	Mean	− 24.156815	− 24.156816	− 24.149847	− 24.052316	− 24.149975	− 24.156815	− 24.156816	− 24.156816
	Max	− 24.156814	− 24.156816	− 24.085134	− 22.99984	− 24.127957	− 24.156815	− 24.156816	− 24.156816
	Std. Dev	3.34E−07	3.553E−15	0.014829	0.2137027	0.0062623	1.565E−07	3.553E−15	3.553E−15
	NFEs	147,665.02	13,320	131,436	150,000	149,950	149,964	8962	138,829
	Rank	5	2	7	8	6	4	2	2
F11	Min	− 43.721918	− 43.721918	− 43.721918	− 43.721862	− 43.721912	− 43.721918	− 43.721918	− 43.721918
	Mean	− 43.721917	− 43.721918	− 43.721918	− 43.718356	− 43.721406	− 43.697423	− 43.721918	− 43.721918
	Max	− 43.721908	− 43.721918	− 43.721918	− 43.695897	− 43.719038	− 42.497173	− 43.721918	− 43.721918
	Std. Dev	1.842E−06	1.421E−14	1.421E−14	0.0052094	0.0005125	0.1714642	1.421E−14	1.421E−14
	NFEs	146,520.6	16,418	5832	150,000	149,950	149,792	5113	116,227
	Rank	5	2.5	2.5	7	6	8	2.5	2.5
F12	Min	− 2.0626119	− 2.0626119	− 2.0626119	− 2.0626119	− 2.0626119	− 2.0626119	− 2.0626119	− 2.0626119
	Mean	− 2.0626119	− 2.0626119	− 2.0626106	− 2.0625604	− 2.06261	− 2.0626119	− 2.0626119	− 2.0626119
	Max	− 2.0626119	− 2.0626119	− 2.0626013	− 2.0622999	− 2.0626045	− 2.0626119	− 2.0626119	− 2.0626119
	Std. Dev	2.464E−09	1.332E−15	1.913E−06	7.927E−05	1.7E−06	7.389E−10	1.332E−15	1.332E−15
	NFEs	150,773.56	21,678	150,000	150,000	149,950	150,000	8141	135,404
	Rank	5	2	6	8	7	4	2	2
F13	Min	2.256E−09	0	0	0	2.986E−06	1.393E−09	0	0
	Mean	1.258E−07	0	0	0.2884609	0.000135	1.557E−07	0	0
	Max	8.524E−07	0	0	0.5637621	0.000692	1.302E−06	0	0
	Std. Dev	1.529E−07	0	0	0.24578	0.0001376	2.096E−07	0	0
	NFEs	150,830.58	56,178	66,049	145,121	149,950	150,000	12,426	141,181
	Rank	5	2.5	2.5	8	7	6	2.5	2.5
F14	Min	2	0	2	0	6.925E−05	1.59E−06	0	0
	Mean	2	1.4	2	1.0003941	0.1129462	1.7600006	0.7376044	8.219E−05
	Max	2	2	2	2.0022288	2.0015164	2.0000001	2	0.0009885
	Std. Dev	9.45E−09	0.9165151	0	1.0000166	0.3869625	0.6499214	0.952814	0.0002148
	NFEs	150,732.8	122,584	150,000	149,625	149,950	150,000	117,111	139,506
	Rank	8	5	7	4	2	6	3	1
F15	Min	− 24,776.518	− 24,776.518	− 24,776.518	− 24,776.518	− 24,776.518	− 24,776.518	− 24,776.518	− 24,776.518
	Mean	− 24,776.518	− 24,776.518	− 24,776.509	− 24,776.518	− 24,776.518	− 24,776.518	− 24,776.518	− 24,776.518
	Max	− 24,776.517	− 24,776.518	− 24,776.465	− 24,776.518	− 24,776.516	− 24,776.516	− 24,776.518	− 24,776.518
	Std. Dev	0.0004613	0	0.0143299	1.474E− 05	0.0003663	0.0003303	0	0
	NFEs	150,828.94	38,798	150,000	150,000	149,950	150,000	18,586	131,767
	Rank	7	2	8	4	5	6	2	2
F16	Min	7.608E−09	0	0	0	0	7.737E−10	0	0
	Mean	8.682E−08	0	0	0	0	2.438E−08	0	0
	Max	3.304E−07	0	0	0	0	1.566E−07	0	0
	Std. Dev	7.742E−08	0	0	0	0	2.871E−08	0	0
	NFEs	150,840.64	24,730	8163	19,238	4424	150,000	8874	1204
	Rank	8	3.5	3.5	3.5	3.5	7	3.5	3.5
F17	Min	0.0644704	0.0644704	0.0644704	0.0644704	0.0644705	0.0644704	0.0644704	0.0644704
	Mean	0.0644704	0.0644704	0.0644704	0.0645096	0.0644725	0.0644704	0.0644704	0.0644704
	Max	0.0644704	0.0644704	0.0644704	0.0648164	0.0644819	0.0644704	0.0644704	0.0644704
	Std. Dev	1.121E−10	4.163E−17	4.163E−17	7.195E−05	2.324E−06	4.378E−11	4.163E−17	4.163E−17
	NFEs	150,790.36	17,910	3848	150,000	149,950	145,984	5477	135,307
	Rank	6	2.5	2.5	8	7	5	2.5	2.5
F18	Min	− 166.02908	− 166.02908	− 166.02905	− 166.027	− 166.02862	− 166.02908	− 166.02908	− 166.02908
	Mean	− 166.02904	− 166.02908	− 165.76174	− 165.84605	− 165.96834	− 166.02907	− 166.02908	− 166.02908
	Max	− 166.02894	− 166.02908	− 163.68494	− 165.35222	− 165.77492	− 166.02906	− 166.02908	− 166.02908
	Std. Dev	3.34E−05	1.421E−13	0.4400681	0.1512756	0.0597362	6.074E−06	1.421E−13	1.421E−13
	NFEs	150,726.86	102,932	150,000	150,000	149,950	150,000	16,488	139,180
	Rank	5	2	8	7	6	4	2	2
F19	Min	8.97E−10	0	6.545E−07	0	2.122E−05	1.37E−10	0	0
	Mean	1.242E−07	0	0.0006953	0.0002875	0.0014032	2.735E−08	0	1.875E−11
	Max	9.183E−07	0	0.0084477	0.0044577	0.0057418	8.83E−08	0	9.375E−10
	Std. Dev	1.482E−07	0	0.0014105	0.0007401	0.0012568	2.424E−08	0	1.313E−10
	NFEs	150,582.74	41,850	150,000	149,395	149,950	150,000	10,199	122,546
	Rank	5	1.5	7	6	8	4	1.5	3
F20	Min	− 2.3458	− 2.3458	− 2.3458	− 2.3458	− 2.3458	− 2.3458	− 2.3458	− 2.3458
	Mean	− 2.3458	− 2.3458	− 2.3458	− 2.3450305	− 2.3457621	− 2.3458	− 2.3458	− 2.3458
	Max	− 2.3458	− 2.3458	− 2.3458	− 2.3407836	− 2.3455475	− 2.3458	− 2.3458	− 2.3458
	Std. Dev	2.22E−15	2.22E−15	2.22E−15	0.0012431	4.487E−05	2.22E−15	2.22E−15	2.22E−15
	NFEs	1183.62	4432	1352	117,396	139,938	76,556	1318	20,966
	Rank	3.5	3.5	3.5	8	7	3.5	3.5	3.5
F21	Min	124.36218	124.36218	124.36218	124.36221	124.36231	124.36218	124.36218	124.36218
	Mean	124.36218	124.36218	124.36218	124.38767	124.37351	124.36218	124.36218	124.36218
	Max	124.36219	124.36218	124.36218	124.58135	124.42111	124.36218	124.36218	124.36218
	Std. Dev	1.271E−06	0	0	0.0410122	0.012138	5.607E−07	0	0
	NFEs	150,859.64	31,454	9163	150,000	149,950	150,000	11,813	134,081
	Rank	6	2.5	2.5	8	7	5	2.5	2.5
F22	Min	− 0.6736675	− 0.6736675	− 0.6736675	− 0.6736675	− 0.6736675	− 0.6736675	− 0.6736675	− 0.6736675
	Mean	− 0.6736675	− 0.6736675	− 0.6736675	− 0.6736662	− 0.6736675	− 0.6736675	− 0.6736675	− 0.6736675
	Max	− 0.6736675	− 0.6736675	− 0.6736673	− 0.673659	− 0.6736675	− 0.6736675	− 0.6736675	− 0.6736675
	Std. Dev	2.815E−13	4.441E−16	3.797E−08	1.86E−06	3.901E−10	5.731E−11	4.441E−16	4.441E−16
	NFEs	73,580.58	14,804	144,236	150,000	146,140	144,058	26,362	106,399
	Rank	4	2	7	8	6	5	2	2
F23	Min	5.878E−12	0	0	0	3.668E−07	2.803E−11	0	0
	Mean	2.067E−09	0	0	0.1709006	4.914E−05	1.714E−09	0	0
	Max	7.778E−09	0	0	0.6522596	0.0002054	1.241E−08	0	0
	Std. Dev	2.05E−09	0	0	0.2092172	4.871E−05	2.322E−09	0	0
	NFEs	150,802.1	25,674	36,540	147,146	149,950	150,000	9245	140,532
	Rank	6	2.5	2.5	8	7	5	2.5	2.5
F24	Min	− 176.54179	− 176.54179	− 176.53511	− 176.53776	− 176.54098	− 176.54179	− 176.54179	− 176.54179
	Mean	− 176.54176	− 176.54179	− 176.39606	− 176.08486	− 176.46763	− 176.54179	− 176.54179	− 176.54179
	Max	− 176.5417	− 176.54179	− 175.40617	− 174.34907	− 175.85529	− 176.54177	− 176.54179	− 176.54179
	Std. Dev	2.566E−05	1.705E−13	0.2056244	0.4274551	0.117937	6.242E−06	1.705E−13	1.705E−13
	NFEs	150,625.4	150,000	150,000	150,000	149,950	150,000	150,000	150,000
	Rank	5	2	7	8	6	4	2	2
F25	Min	− 176.13757	− 176.13757	− 176.13706	− 176.13349	− 176.13666	− 176.13757	− 176.13757	− 176.13757
	Mean	− 176.13756	− 176.13757	− 176.07398	− 175.90307	− 176.10669	− 153.47544	− 176.13757	− 176.13757
	Max	− 176.13752	− 176.13757	− 175.73141	− 174.87606	− 176.04619	− 90.885324	− 176.13757	− 176.13757
	Std. Dev	1.553E−05	2.842E−14	0.0854863	0.2178573	0.0257696	25.579229	2.842E−14	2.842E−14
	NFEs	144,434.6	26,452	150,000	150,000	149,950	148,756	4660	103,167
	Rank	4	2	6	7	5	8	2	2
F26	Min	6.313E−12	0	0	0	0	0	0	0
	Mean	1.376E−09	0	0	0	0	4.688E−10	0	0
	Max	4.753E−09	0	0	0	0	2.147E−09	0	0
	Std. Dev	1.083E−09	0	0	0	0	4.674E−10	0	0
	NFEs	150,790.52	16,980	10,538	18,943	6404	150,000	6662	1388
	Rank	8	3.5	3.5	3.5	3.5	7	3.5	3.5
F27	Min	− 1.913223	− 1.913223	− 1.9132222	− 1.9132229	− 1.9132228	− 1.913223	− 1.913223	− 1.913223
	Mean	− 1.913223	− 1.913223	− 1.9132117	− 1.913222	− 1.9132114	− 1.913223	− 1.913223	− 1.913223
	Max	− 1.9132229	− 1.913223	− 1.9131955	− 1.9132168	− 1.9131623	− 1.913223	− 1.913223	− 1.913223
	Std. Dev	1.417E−09	2.22E−15	7.228E−06	1.149E−06	1.198E−05	6.072E−10	2.22E−15	2.22E−15
	NFEs	150,796.96	18,484	150,000	150,000	149,950	150,000	6804	122,920
	Rank	5	2	7	6	8	4	2	2
F28	Min	− 19.966676	− 19.966682	− 19.966682	− 19.966446	− 19.965824	− 19.966682	− 19.966682	− 19.966682
	Mean	− 19.966614	− 19.966682	− 19.966682	− 19.9516	− 19.959177	− 19.966637	− 19.966682	− 19.966682
	Max	− 19.966544	− 19.966682	− 19.966682	− 19.873952	− 19.948416	− 19.966578	− 19.966682	− 19.966682
	Std. Dev	3.598E−05	3.553E−15	3.553E−15	0.0164593	0.0044158	2.366E−05	3.553E−15	3.553E−15
	NFEs	150,724.42	26,880	6058	150,000	149,950	150,000	7125	135,832
	Rank	6	2.5	2.5	8	7	5	2.5	2.5
F29	Min	− 1.8013	− 1.8013	− 1.8013	− 1.7308521	− 1.801276	− 1.8013	− 1.8013	− 1.8013
	Mean	− 1.8013	− 1.8013	− 1.8013	− 1.2553068	− 1.7366044	− 1.8013	− 1.8013	− 1.8013
	Max	− 1.8013	− 1.8013	− 1.8013	− 0.9999082	− 1	− 1.8013	− 1.8013	− 1.8013
	Std. Dev	1.11E−15	1.11E−15	1.11E−15	0.2350477	0.2172136	1.11E−15	1.11E−15	1.11E−15
	NFEs	9391.16	7936	3199	150,000	149,950	135,100	2040	66,819
	Rank	3.5	3.5	3.5	8	7	3.5	3.5	3.5
F30	Min	− 1.0198295	− 1.0198295	− 1.0198295	− 1.0198295	− 1.0198295	− 1.0198295	− 1.0198295	− 1.0198295
	Mean	− 1.0192414	− 1.0198295	− 1.0198295	− 1.0198295	− 1.0198295	− 1.0198295	− 1.0198295	− 1.0198295
	Max	− 1.0100283	− 1.0198295	− 1.0198295	− 1.0198295	− 1.0198294	− 1.0198295	− 1.0198295	− 1.0198295
	Std. Dev	0.0023277	8.882E−16	1.447E−09	1.28E−09	1.92E−08	1.682E−11	3.994E−12	8.882E−16
	NFEs	85,641.02	12,278	148,441	130,001	149,950	146,495	148,832	87,002
	Rank	8	1.5	6	5	7	4	3	1.5
F31	Min	− 2.2839498	− 2.2839498	− 2.2839498	− 2.2839494	− 2.2839466	− 2.2839498	− 2.2839498	− 2.2839498
	Mean	− 2.2587729	− 2.2839498	− 2.2839498	− 2.2839256	− 2.2837873	− 2.2839498	− 2.2839498	− 2.2839498
	Max	− 1.8643355	− 2.2839498	− 2.2839498	− 2.2837646	− 2.2832559	− 2.2839498	− 2.2839498	− 2.2839498
	Std. Dev	0.0996529	2.22E−15	2.22E−15	3.073E−05	0.0001718	1.146E−08	2.22E−15	2.22E−15
	NFEs	150,757.1	31,624	10,498	150,000	149,950	150,000	8584	129,308
	Rank	8	2.5	2.5	6	7	5	2.5	2.5
F32	Min	1.693E−11	0	0	0	2.228E−07	5.213E−12	0	0
	Mean	1.395E−08	0	0	6.251E−05	0.0001543	6.871E−07	0	0
	Max	8.348E−08	0	0	0.0017811	0.0017885	3.21E−05	0	0
	Std. Dev	1.689E−08	0	0	0.0002739	0.0003112	4.489E−06	0	0
	NFEs	150,827.5	15,354	16,216	114,005	149,950	150,000	7777	123,591
	Rank	5	2.5	2.5	7	8	6	2.5	2.5
F33	Min	− 0.9635348	− 0.9635348	− 0.9635348	− 0.9635348	− 0.9635346	− 0.9635348	− 0.9635348	− 0.9635348
	Mean	− 0.9635348	− 0.9635348	− 0.9635298	− 0.9634256	− 0.9635281	− 0.9635348	− 0.9635348	− 0.9635348
	Max	− 0.9635348	− 0.9635348	− 0.9634769	− 0.9625693	− 0.963507	− 0.9635348	− 0.9635348	− 0.9635348
	Std. Dev	4.176E−10	9.992E−16	1.056E−05	0.000185	5.323E−06	1.029E−10	9.992E−16	9.992E−16
	NFEs	150,679.16	24,098	142,413	150,000	149,950	150,000	8536	137,509
	Rank	5	2	6	8	7	4	2	2
F34	Min	0.9	0.9	0.9	0.9	0.9	0.9	0.9	0.9
	Mean	0.904	0.9	0.9043351	0.9	0.9	0.94	0.9	0.9
	Max	1	0.9	1.0000002	0.9	0.9	1	0.9	0.9
	Std. Dev	0.0195959	1.011E−11	0.0195332	8.882E−16	8.882E−16	0.0489898	8.882E−16	8.882E−16
	NFEs	150,584.64	92,474	148,689	18,937	6074	150,000	9459	1642
	Rank	6	5	7	2.5	2.5	8	2.5	2.5
F35	Min	1.083E−05	0	2.269E−09	0	2.515E−06	1.533E−08	0	0
	Mean	7.2E−05	0	0.0001567	0.0296118	0.000278	7.895E−06	0	0
	Max	0.0002798	0	0.0015145	0.4464966	0.0012055	2.865E−05	0	0
	Std. Dev	5.903E−05	0	0.0002774	0.0751466	0.0002447	7.971E−06	0	0
	NFEs	150,634.56	33,662	150,000	147,196	149,950	150,000	15,439	121,730
	Rank	5	2	6	8	7	4	2	2
F36	Min	0.9	0.9	0.9	0.9	0.9	0.9	0.9	0.9
	Mean	0.902	0.9	0.9022549	0.9	0.9	0.956	0.9	0.9
	Max	1.0000001	0.9	1.0000188	0.9	0.9	1	0.9	0.9
	Std. Dev	0.014	8.882E−16	0.013969	8.882E−16	8.882E−16	0.0496387	8.882E−16	8.882E−16
	NFEs	150,522.18	88,092	149,244	19,153	6038	150,000	10,029	1923
	Rank	6	3	7	3	3	8	3	3
F37	Min	4.882E−10	0	0	0	0	5.268E−09	0	0
	Mean	2.889E−06	0	2.138E−12	0.002424	0	6.38E−06	0	0
	Max	2.071E−05	0	1.52E−11	0.0899116	0	4.405E−05	0	0
	Std. Dev	3.942E−06	0	3.381E−12	0.01277	0	9.844E−06	0	0
	NFEs	150,778.72	43,402	119,868	53,231	46,529	150,000	15,122	6932
	Rank	6	2.5	5	8	2.5	7	2.5	2.5
F38	Min	− 3873.7242	− 3873.7242	− 3873.7242	− 3873.7242	− 3873.7242	− 3873.7242	− 3873.7242	− 3873.7242
	Mean	− 3873.7242	− 3873.7242	− 3873.7242	− 3873.7164	− 3873.724	− 3873.7242	− 3873.7242	− 3873.7242
	Max	− 3873.7242	− 3873.7242	− 3873.7242	− 3873.6565	− 3873.7237	− 3873.7242	− 3873.7242	− 3873.7242
	Std. Dev	2.267E−06	0	0	0.0140267	0.0001222	6.572E−07	0	0
	NFEs	150,831.46	22,814	5346	150,000	149,950	150,000	10,542	129,884
	Rank	6	2.5	2.5	8	7	5	2.5	2.5
F39	Min	− 2.1999998	− 2.2	− 2.2	− 2.1999676	− 2.1993792	− 2.2	− 2.2	− 2.2
	Mean	− 2.1862541	− 2.2	− 2.2	− 2.1873374	− 2.1741647	− 2.1999865	− 2.2	− 2.2
	Max	− 1.878412	− 2.2	− 2.2	− 2.1467488	− 1.197857	− 2.1999189	− 2.2	− 2.2
	Std. Dev	0.0504614	1.332E−15	1.332E−15	0.0102714	0.139499	1.449E−05	1.332E−15	1.332E−15
	NFEs	150,819.32	80,638	49,622	150,000	149,950	150,000	37,427	141,833
	Rank	7	2.5	2.5	6	8	5	2.5	2.5
F40	Min	− 2	− 2	− 2	− 2	− 1.9999978	− 2	− 2	− 2
	Mean	− 1.9966894	− 2	− 2	− 1.9586812	− 1.9999167	− 2	− 2	− 2
	Max	− 1.9172359	− 2	− 2	− 1.5572018	− 1.9994763	− 1.9999998	− 2	− 2
	Std. Dev	0.0162184	0	0	0.0891136	9.768E−05	4.243E−08	0	0
	NFEs	150,866.58	41,076	13,838	150,000	149,950	150,000	14,200	113,798
	Rank	7	2.5	2.5	8	6	5	2.5	2.5
F41	Min	34.040244	34.040243	34.041799	34.040259	34.040261	34.040243	34.040243	34.040243
	Mean	70.004025	35.638635	60.429604	65.44762	34.078729	62.811268	34.040243	34.040545
	Max	74	74	74	74.645055	34.200075	74	34.040243	34.044278
	Std. Dev	11.987926	7.8304808	18.907141	16.334442	0.0360058	17.941886	3.553E−14	0.0007264
	NFEs	150,906.58	68,526	150,000	150,000	149,950	150,000	15,282	135,124
	Rank	8	4	5	7	3	6	1	2
F42	Min	9.509E−07	0	0	0	0	2.153E−06	0	0
	Mean	0.0001767	0	0	0	0	8.035E−05	0	0
	Max	0.0007141	0	0	0	0	0.0003803	0	0
	Std. Dev	0.0001443	0	0	0	0	8.022E−05	0	0
	NFEs	150,888.76	28,920	8148	22,601	7467	150,000	15,459	1643
	Rank	8	3.5	3.5	3.5	3.5	7	3.5	3.5
F43	Min	5.846E−07	0	0	0	0	1.538E−08	0	0
	Mean	2.716E−05	0	0	0	0	1.019E−05	0	0
	Max	0.0001105	0	0	0	0	6.326E−05	0	0
	Std. Dev	2.765E−05	0	0	0	0	1.115E−05	0	0
	NFEs	150,907.68	26,190	7723	21,785	7216	150,000	12,776	1519
	Rank	8	3.5	3.5	3.5	3.5	7	3.5	3.5
F44	Min	8.015E−12	0	0	0	0	9.939E−12	0	0
	Mean	1.081E−09	0	0	0	0	5.018E−10	0	0
	Max	6.221E−09	0	0	0	0	2.927E−09	0	0
	Std. Dev	1.243E−09	0	0	0	0	6.015E−10	0	0
	NFEs	150,924.6	48,132	18,602	25,219	4935	150,000	8204	1374
	Rank	8	3.5	3.5	3.5	3.5	7	3.5	3.5
F45	Min	0.0015669	0.0015669	0.0015669	0.0015669	0.0015669	0.0015669	0.0015669	0.0015669
	Mean	0.0015672	0.0015669	0.0015683	0.0015681	0.0015669	0.0015669	0.0015669	0.0015669
	Max	0.0015687	0.0015669	0.0015749	0.0015763	0.0015672	0.0015674	0.0015669	0.0015669
	Std. Dev	3.142E−07	1.952E−18	1.74E−06	1.673E−06	6.137E−08	1.203E−07	1.952E−18	1.952E−18
	NFEs	150,984.36	119,666	150,000	150,000	149,950	150,000	19,133	116,872
	Rank	6	2	8	7	4	5	2	2
F46	Min	0.292579	0.292579	0.292579	0.292579	0.292579	0.292579	0.292579	0.292579
	Mean	0.292579	0.292579	0.2925793	0.2925794	0.292579	0.292579	0.292579	0.292579
	Max	0.2925793	0.292579	0.2925824	0.2925825	0.292579	0.2925791	0.292579	0.292579
	Std. Dev	4.95E−08	0	6.479E−07	6.471E−07	0	7.565E−09	0	0
	NFEs	97,215.4	36,136	105,259	115,043	46,019	143,035	10,562	41,718
	Rank	6	2.5	7	8	2.5	5	2.5	2.5
F47	Min	− 26.920336	− 26.920336	− 26.920336	− 26.920058	− 26.920305	− 26.920336	− 26.920336	− 26.920336
	Mean	− 26.920335	− 26.920336	− 26.918206	− 26.470796	− 26.916463	− 26.920335	− 26.920336	− 26.920336
	Max	− 26.920334	− 26.920336	− 26.89308	− 24.893102	− 26.901966	− 26.920335	− 26.920336	− 26.920336
	Std. Dev	3.083E−07	1.421E−14	0.0053345	0.4860863	0.0037564	6.892E−08	1.421E−14	1.421E−14
	NFEs	150,898.76	33,114	125,243	150,000	149,950	150,000	15,166	149,650
	Rank	5	2	6	8	7	4	2	2
F48	Min	− 19.2085	− 19.2085	− 19.2085	− 19.208499	− 19.208464	− 19.2085	− 19.2085	− 19.2085
	Mean	− 19.2085	− 19.2085	− 19.182987	− 19.154687	− 19.205055	− 19.2085	− 19.2085	− 19.2085
	Max	− 19.2085	− 19.2085	− 18.020717	− 18.947059	− 19.193237	− 19.2085	− 19.2085	− 19.2085
	Std. Dev	1.421E−14	1.421E−14	0.1661985	0.0633054	0.0036247	1.421E−14	1.421E−14	1.421E−14
	NFEs	60,705.8	8362	121,320	150,000	149,950	146,656	5988	92,717
	Rank	3	3	7	8	6	3	3	3
F49	Min	− 24.156816	− 24.156816	− 24.156816	− 24.156793	− 24.156666	− 24.156816	− 24.156816	− 24.156816
	Mean	− 24.156815	− 24.156816	− 24.152432	− 24.052886	− 24.14902	− 24.156815	− 24.156816	− 24.156816
	Max	− 24.156814	− 24.156816	− 24.043155	− 22.600258	− 24.124724	− 24.156815	− 24.156816	− 24.156816
	Std. Dev	3.643E−07	3.553E−15	0.0179508	0.2545333	0.0070307	7.818E−08	3.553E−15	3.553E−15
	NFEs	149,117.04	13,144	107,835	150,000	149,950	149,981	8845	139,628
	Rank	5	2	6	8	7	4	2	2
F50	Min	− 4.8168141	− 4.8168141	− 4.8168141	− 4.816814	− 4.8168141	− 4.8168141	− 4.8168141	− 4.8168141
	Mean	− 4.8168141	− 4.8168141	− 4.8168141	− 4.8168115	− 4.8168141	− 4.8168141	− 4.8168141	− 4.8168141
	Max	− 4.8168141	− 4.8168141	− 4.8168141	− 4.816804	− 4.816814	− 4.8168141	− 4.8168141	− 4.8168141
	Std. Dev	1.122E−09	2.665E−15	2.665E−15	2.315E−06	1.291E−08	6.057E−10	2.665E−15	2.665E−15
	NFEs	150,767.72	17,856	4531	150,000	149,950	150,000	6525	121,391
	Rank	6	2.5	2.5	8	7	5	2.5	2.5
F51	Min	− 2.9999986	− 3	− 3	− 3	− 3	− 2.9999992	− 3	− 3
	Mean	− 2.9999711	− 3	− 3	− 3	− 3	− 2.999984	− 3	− 3
	Max	− 2.9999165	− 3	− 3	− 3	− 3	− 2.9999597	− 3	− 3
	Std. Dev	1.635E−05	0	0	0	0	8.645E−06	0	0
	NFEs	150,829.94	50,204	11,902	21,641	10,312	150,000	40,795	1994
	Rank	8	3.5	3.5	3.5	3.5	7	3.5	3.5
F52	Min	− 1.4999956	− 1.5	− 1.5	− 1.5	− 1.5	− 1.4999989	− 1.5	− 1.5
	Mean	− 1.4999854	− 1.5	− 1.5	− 1.5	− 1.5	− 1.4999912	− 1.5	− 1.5
	Max	− 1.4999622	− 1.5	− 1.5	− 1.5	− 1.5	− 1.4999806	− 1.5	− 1.5
	Std. Dev	7.78E−06	0	0	0	0	4.229E−06	0	0
	NFEs	150,837.02	53,750	12,375	21,376	10,728	150,000	37,349	2010
	Rank	8	3.5	3.5	3.5	3.5	7	3.5	3.5
F53	Min	− 8.5536	− 8.5536	− 8.5536	− 8.5536	− 8.5536	− 8.5536	− 8.5536	− 8.5536
	Mean	− 8.1956153	− 8.5536	− 8.5172819	− 8.5536	− 8.5536	− 7.9079136	− 8.3987104	− 8.5536
	Max	− 6.4126404	− 8.5536	− 7.6456102	− 8.5536	− 8.5536	− 5.574845	− 7.645779	− 8.5536
	Std. Dev	0.5499427	5.329E−15	0.1779217	5.329E−15	5.329E−15	0.6449876	0.3266286	5.329E−15
	NFEs	52,712.52	370	6582	5343	55	93,088	150,000	92
	Rank	7	2.5	5	2.5	2.5	8	6	2.5
F54	Min	− 400	− 400	− 400	− 400	− 400	− 400	− 400	− 400
	Mean	− 400	− 400	− 400	− 400	− 400	− 400	− 400	− 400
	Max	− 400	− 400	− 400	− 400	− 400	− 400	− 400	− 400
	Std. Dev	3.272E−07	0	0	0	0	1.23E−07	0	0
	NFEs	150,916.24	28,970	8418	12,097	5499	150,000	9981	1269
	Rank	8	3.5	3.5	3.5	3.5	7	3.5	3.5
F55	Min	19.105881	19.10588	19.10588	19.105921	19.105914	19.105881	19.10588	19.10588
	Mean	19.106095	19.10588	19.10588	21.831613	19.109977	19.105978	19.10588	19.10588
	Max	19.106738	19.10588	19.10588	32.43579	19.12056	19.106313	19.10588	19.10588
	Std. Dev	0.0001957	1.776E−14	1.776E−14	3.0751262	0.0035102	0.0001013	1.776E−14	1.776E−14
	NFEs	150,774.86	31,054	6488	150,000	149,950	150,000	15,750	132,953
	Rank	6	2.5	2.5	8	7	5	2.5	2.5
F56	Min	− 0.0037912	− 0.0037912	− 0.0037912	− 0.0037912	− 0.0037912	− 0.0037912	− 0.0037912	− 0.0037912
	Mean	− 0.0037912	− 0.0037912	− 0.0037912	− 0.0037912	− 0.0037912	− 0.0037912	− 0.0037912	− 0.0037912
	Max	− 0.0037912	− 0.0037912	− 0.0037912	− 0.0037912	− 0.0037912	− 0.0037912	− 0.0037912	− 0.0037912
	Std. Dev	8.126E−10	5.204E−18	2.177E−10	5.204E−18	1.589E−11	3.115E−10	5.204E−18	5.204E−18
	NFEs	150,954	21,796	150,000	38,727	133,528	149,998	7320	115,358
	Rank	8	2.5	6	2.5	5	7	2.5	2.5
F57	Min	− 0.3523861	− 0.3523861	− 0.3523861	− 0.3523861	− 0.3523861	− 0.3523861	− 0.3523861	− 0.3523861
	Mean	− 0.3523861	− 0.3523861	− 0.352386	− 0.3523861	− 0.3523861	− 0.3523861	− 0.3523861	− 0.3523861
	Max	− 0.352386	− 0.3523861	− 0.3523858	− 0.3523861	− 0.3523861	− 0.3523861	− 0.3523861	− 0.3523861
	Std. Dev	9.064E−09	1.11E−16	6.427E−08	4.061E−12	2.41E−09	3.554E−09	1.11E−16	1.11E−16
	NFEs	150,749.54	19,846	150,000	117,831	149,950	150,000	7571	123,275
	Rank	7	2	8	4	5	6	2	2
F58	Min	4.93E−07	0	0	0	0	1.391E−07	0	0
	Mean	3.532E−05	0	0	0	0	1.165E−05	0	0
	Max	0.0001322	0	0	0	0	6.095E−05	0	0
	Std. Dev	3.135E−05	0	0	0	0	1.326E−05	0	0
	NFEs	150,907.54	26,578	7152	21,851	6198	150,000	13,437	1399
	Rank	8	3.5	3.5	3.5	3.5	7	3.5	3.5
F59	Min	0	0	0	0	0	0	0	0
	Mean	0.0285544	0	0	0	0	0.2326575	0	0
	Max	0.187125	0	0	0	0	1.318125	0	0
	Std. Dev	0.033247	0	0	0	0	0.3752315	0	0
	NFEs	150,564.48	36,892	13,879	27,583	11,402	149,915	7623	1296
	Rank	7	3.5	3.5	3.5	3.5	8	3.5	3.5
F60	Min	− 9.6538418	− 9.5216433	− 8.5956837	− 9.0184572	− 5.3826485	− 9.1580302	− 9.5723648	− 9.66015
	Mean	− 9.2719934	− 9.1090113	− 7.0141044	− 8.2878871	− 4.1724485	− 7.2714165	− 8.8163225	− 9.6116718
	Max	− 8.7413045	− 8.5331766	− 5.5373408	− 7.2030572	− 3.1441789	− 5.0650679	− 7.4330183	− 9.4333188
	Std. Dev	0.2026321	0.1935088	0.7028076	0.3683613	0.4858631	0.8917418	0.5197141	0.0477421
	NFEs	150,783.96	150,000	150,000	150,000	149,950	150,000	150,000	149,386
	Rank	2	3	7	5	8	6	4	1

**Table 6:** Comparative results of algorithms for the *N*-dimensional functions.

No	Statistics	Methods							
		FA	CS	Jaya	TEO	SCA	MVO	CSA	FuFiO
F61	Min	0.1909505	2.5048585	4.702E−08	0	1.069E−11	0.0294228	0.0001546	0
	Mean	0.2357005	6.8287435	11.015686	0	17.879896	0.32013	3.1587468	0
	Max	0.2789645	13.377361	19.979473	0	20.316789	2.1249327	5.4122706	0
	Std. Dev	0.0200211	2.6266095	9.7270521	0	6.2815761	0.5385861	0.88528	0
	NFEs	150,604.36	150,000	150,000	31,224	149,950	150,000	150,000	8594
	Rank	3	6	7	1.5	8	4	5	1.5
F62	Min	0.0293937	3.6621256	6.633E−06	0	0	0.6831033	0.0019468	0
	Mean	0.1083397	5.4183604	2.0682974	0	0.0189388	2.0075273	0.0731469	0
	Max	0.3514523	7.2910447	17.307255	0	0.7953306	6.1175488	1.0803344	0
	Std. Dev	0.0725235	0.8871063	4.1886493	0	0.1116362	1.1861554	0.1739759	0
	NFEs	150,643	150,000	150,000	31,001	124,709	150,000	150,000	7906
	Rank	5	8	7	1.5	3	6	4	1.5
F63	Min	0.000325	8.162E−09	0	0	0	2.379E−05	1.494E−08	0
	Mean	0.0004231	2.783E−08	0	0	0	5.185E−05	1.593E−07	0
	Max	0.0005437	7.79E−08	0	0	0	9.892E−05	1.051E−06	0
	Std. Dev	5.154E−05	1.69E−08	0	0	0	1.452E−05	2.173E−07	0
	NFEs	150,634.18	150,000	107,113	22,542	78,925	150,000	150,000	4242
	Rank	8	5	2.5	2.5	2.5	7	6	2.5
F64	Min	0.0882958	2.236E−12	0	0	0	0.0001167	0	0
	Mean	0.139256	4.206E−11	0	0	0	0.0003964	0	0
	Max	0.2302019	2.036E−10	0	0	0	0.0012738	0	0
	Std. Dev	0.0288142	3.687E−11	0	0	0	0.0002286	0	0
	NFEs	150,537.44	150,000	85,597	20,429	74,301	150,000	116,899	3313
	Rank	8	6	3	3	3	7	3	3
F65	Min	− 2.8407777	− 2.7094506	− 2.5377361	− 3	− 3	− 2.666686	− 3	− 3
	Mean	− 2.6938899	− 2.5681796	− 1.6645717	− 2.1014467	− 2.9990427	− 2.3279867	− 2.8593801	− 2.9909064
	Max	− 2.4757505	− 2.4814892	− 1.0744921	− 1.8262406	− 2.9704144	− 2.0318231	− 2.5873465	− 2.8563701
	Std. Dev	0.0817029	0.0457589	0.4015555	0.220334	0.0047897	0.1291195	0.0933584	0.030772
	NFEs	150,561.02	150,000	150,000	147,400	99,640	150,000	150,000	31,429
	Rank	4	5	8	7	1	6	3	2
F66	Min	0	0	0	0	0	0	0	0
	Mean	0	0	0	0	0	0	0	0
	Max	0	0	0	0	0	0	0	0
	Std. Dev	0	0	0	0	0	0	0	0
	NFEs	83,603.08	102,576	57,227	12,501	75,047	145,323	43,363	1432
	Rank	4.5	4.5	4.5	4.5	4.5	4.5	4.5	4.5
F67	Min	− 0.9987171	− 0.9864462	− 0.6059982	− 0.9351795	− 0.6515629	− 0.999978	− 1	− 0.999924
	Mean	− 0.9797723	− 0.9795079	− 0.5695104	− 0.8093916	− 0.5946674	− 0.9986162	− 0.9476279	− 0.996766
	Max	− 0.8934949	− 0.9722801	− 0.5303084	− 0.6038427	− 0.5261549	− 0.9666056	− 0.8666668	− 0.9906059
	Std. Dev	0.0276643	0.0031058	0.0171687	0.075034	0.0287077	0.0065319	0.0360735	0.0024981
	NFEs	150,240.18	150,000	150,000	150,000	149,950	150,000	150,000	150,000
	Rank	3	4	8	6	7	1	5	2
F68	Min	− 0.9998249	− 0.9956164	− 0.8916387	− 0.6729154	− 0.5968834	− 0.9999779	− 0.9999999	− 0.9999298
	Mean	− 0.9997161	− 0.9934403	− 0.6557599	− 0.5946617	− 0.5353036	− 0.9927801	− 0.945119	− 0.997763
	Max	− 0.9994418	− 0.990737	− 0.5843903	− 0.5018163	− 0.456915	− 0.9665975	− 0.866756	− 0.9937146
	Std. Dev	8.536E−05	0.001254	0.0638435	0.031871	0.0317675	0.0135061	0.0321861	0.0016216
	NFEs	150,687.86	150,000	150,000	150,000	149,950	150,000	150,000	150,000
	Rank	1	3	6	7	8	4	5	2
F69	Min	0.7071839	0.6667536	0	0.6666667	0.6666777	0.6683365	0.6666811	0.6666667
	Mean	0.7198196	0.6697147	0.6001154	0.6685618	0.6668629	0.8042692	0.7277258	0.6666667
	Max	0.743307	0.6877307	0.6724336	0.7201022	0.669328	1.4526621	1.3447683	0.6666667
	Std. Dev	0.0069476	0.003796	0.2000401	0.0075704	0.0003774	0.1707243	0.1265789	1.264E−08
	NFEs	150,654.28	150,000	148,870	150,000	149,950	150,000	150,000	150,000
	Rank	6	5	1	4	3	8	7	2
F70	Min	− 0.9999857	− 1	− 1	− 1	− 1	− 0.9999995	− 1	− 1
	Mean	− 0.9999811	− 1	− 1	− 1	− 1	− 0.9999991	− 1	− 1
	Max	− 0.9999775	− 1	− 1	− 1	− 1	− 0.9999986	− 1	− 1
	Std. Dev	1.734E−06	1.353E−10	0	0	0	2.571E−07	1.493E−12	0
	NFEs	150,601.14	150,000	98,814	18,432	77,604	150,000	149,190	3834
	Rank	8	6	2.5	2.5	2.5	7	5	2.5
F71	Min	0.0136628	9.762E−06	0	0	0	0.0009591	1.381E−07	0
	Mean	0.0205724	0.0005598	0.083938	0	0.0269116	0.0186794	0.0123858	0
	Max	0.0371669	0.0057166	0.3590051	0	0.757095	0.0454063	0.0663026	0
	Std. Dev	0.0038833	0.0009266	0.1002033	0	0.1123686	0.0116117	0.0172719	0
	NFEs	150,687.56	150,000	147,427	23,475	115,725	150,000	150,000	4813
	Rank	6	3	8	1.5	7	5	4	1.5
F72	Min	9.07E−06	0	0	0	0	8.696E−09	0	0
	Mean	1.662E−05	1.13E−11	0	0	0	9.153E−08	0	0
	Max	3.205E−05	2.011E−10	0	0	0	2.951E−07	0	0
	Std. Dev	4.734E−06	2.859E−11	0	0	0	6.492E−08	0	0
	NFEs	150,537.58	149,604	90,679	17,985	84,676	150,000	106,382	2831
	Rank	8	6	3	3	3	7	3	3
F73	Min	0.0237561	24.963929	0	45.437113	66.145956	0.5784322	8.8575699	0.2291156
	Mean	7.291771	74.586736	0.1739121	77.633327	85.57113	70.587575	29.462289	4.4853881
	Max	121.05228	157.9981	3.636199	121.52253	127.36365	451.62605	55.797938	13.687722
	Std. Dev	28.737176	27.106377	0.5249535	17.296893	11.192779	104.26181	10.980962	2.4950105
	NFEs	150,658.06	150,000	146,317	150,000	149,950	150,000	150,000	150,000
	Rank	3	6	1	7	8	5	4	2
F74	Min	2.0028938	2	2	9.3218549	9,481,483.9	2	603.45497	2
	Mean	2.0063296	2.0000048	2	16.578871	9.73E + 10	2.0164954	9,470,382.4	2.0035558
	Max	2.0090752	2.0001172	2	27.052386	2.785E + 12	2.0749961	188,217,730	2.0892947
	Std. Dev	0.0013636	1.788E−05	0	4.3674552	4.037E + 11	0.016127	32,131,011	0.0174199
	NFEs	150,549.66	148,552	1445	150,000	149,950	147,522	150,000	13,494
	Rank	4	2	1	6	8	5	7	3
F75	Min	2.004438	2	2	10.919907	60,449,472	2.0079165	1575.3435	2
	Mean	2.0066882	2.0000056	2	17.149951	4.364E + 10	2.1104896	30,189,258	2.0041156
	Max	2.0087217	2.0000802	2	24.729305	5.059E + 11	3.5331902	1.009E + 09	2.1463304
	Std. Dev	0.0010125	1.387E−05	0	4.0710945	1.03E + 11	0.2288759	142,182,139	0.0208942
	NFEs	150,664.7	149,644	1398	150,000	149,950	150,000	150,000	31,932
	Rank	4	2	1	6	8	5	7	3
F76	Min	0	0	0	0	0	0	0	0
	Mean	1.623E−11	0	3.676E−08	1.889E−09	1.983E−09	7.385E−11	0	3.374E−08
	Max	1.457E−10	0	4.299E−07	6.636E−08	1.751E−08	6.475E−10	0	8.967E−07
	Std. Dev	2.994E−11	0	8.354E−08	9.652E−09	3.468E−09	1.163E−10	0	1.369E−07
	NFEs	126,613.98	27,150	148,361	51,591	149,691	146,959	15,877	86,978
	Rank	3	1.5	8	5	6	4	1.5	7
F77	Min	3.49E−11	0	0	0	0.0001266	0	0	0
	Mean	9.189E−11	5.752E−09	0	0	0.004732	0	0	3.196E−09
	Max	1.67E−10	2.435E−07	0	0	0.0160116	0	0	1.293E−07
	Std. Dev	3.201E−11	3.442E−08	0	0	0.0035257	0	0	1.836E−08
	NFEs	150,623.84	34,716	4132	27,056	149,950	149,249	52,038	11,086
	Rank	5	7	2.5	2.5	8	2.5	2.5	6
F78	Min	1.487E−09	0	1.73E−08	0	9.073E−09	5.983E−11	1.028E−10	0
	Mean	1.253E−07	2.251E−09	7.412E−06	1.484E−08	6.637E−06	5.372E−08	1.303E−08	2.003E−06
	Max	8.5E−07	2.078E−08	3.952E−05	1.36E−07	4.681E−05	4.187E−07	1.233E−07	2.988E−05
	Std. Dev	1.678E−07	3.837E−09	9.458E−06	2.872E−08	9.741E−06	7.402E−08	2.356E−08	5.344E−06
	NFEs	150,560.56	148,544	150,000	148,948	149,950	150,000	150,000	146,781
	Rank	5	1	8	3	7	4	2	6
F79	Min	4.75035	714.16445	9.08E−10	0	0	329.97758	8.2728704	0
	Mean	342.71711	1551.7937	194.12301	0	2.956E−14	1353.2583	510.95862	0
	Max	1865.3502	2221.5937	1780.8772	0	1.478E−12	3311.3709	1429.6684	0
	Std. Dev	389.49659	375.28206	300.40704	0	2.069E−13	595.55732	382.41572	0
	NFEs	150,648.18	150,000	150,000	25,900	91,836	150,000	150,000	5557
	Rank	5	8	4	1.5	3	7	6	1.5
F80	Min	4.277E−08	0	8.666E−10	0	0	1.08E−08	0	0
	Mean	5.203E−07	0	5.534E−08	0	8.773E−10	1.43E−06	0	9.974E−12
	Max	2.146E−06	0	2.445E−07	0	2.574E−08	6.952E−06	0	2.55E−10
	Std. Dev	3.919E−07	0	5.364E−08	0	3.895E−09	1.62E−06	0	3.898E−11
	NFEs	150,816.74	28,890	150,000	73,509	104,644	150,000	17,268	62,776
	Rank	7	2	6	2	5	8	2	4
F81	Min	0.0507989	1.904E−06	8.42E−08	0	0	0.0063994	0.0087553	0
	Mean	0.0704927	5.177E−06	2.343E−06	0	6.602E−10	0.0555153	0.0945922	0
	Max	0.08765	9.856E−06	1.679E−05	0	2.908E−08	0.1425746	0.2030205	0
	Std. Dev	0.0081709	1.886E−06	3.075E−06	0	4.093E−09	0.0364778	0.0483201	0
	NFEs	150,583.88	150,000	150,000	24,305	100,247	150,000	150,000	4937
	Rank	7	5	4	1.5	3	6	8	1.5
F82	Min	7.284E−11	0	0	0	0	1.928E−09	3.676E−12	0
	Mean	8.477E−10	0	0	0	0	1.48E−08	1.512E−10	0
	Max	4.974E−09	0	0	0	0	4.162E−08	6.752E−10	0
	Std. Dev	8.408E−10	0	0	0	0	8.686E−09	1.462E−10	0
	NFEs	150,509.1	58,536	45,735	17,035	74,357	150,000	150,000	1397
	Rank	7	3	3	3	3	8	6	3
F83	Min	16.206529	57.220652	118.2245	0	0	54.732461	8.9546315	0
	Mean	39.402389	73.375657	198.48474	0	6.6754191	101.73463	20.874231	3.4557393
	Max	64.29964	97.351438	252.42519	0	91.501502	150.24768	55.717622	22.052833
	Std. Dev	11.233519	9.0611894	23.626837	0	21.689954	21.638388	10.167064	6.4356794
	NFEs	150,798.86	150,000	150,000	30,788	126,313	150,000	150,000	50,853
	Rank	5	6	8	1	3	7	4	2
F84	Min	476.83692	16.95717	6.2683203	2469.6739	3333.2051	21.138824	0.0125266	17.659501
	Mean	848.38134	34.174575	616.30913	4359.35	4382.0782	72.037714	0.6154346	857.28865
	Max	1339.9647	84.140641	1743.4195	6880.4811	5528.4971	281.59346	8.7294869	2146.8334
	Std. Dev	167.28326	12.072643	451.86434	950.17063	513.79574	44.536416	1.4947032	472.1903
	NFEs	150,642.12	150,000	150,000	150,000	149,950	150,000	150,000	150,000
	Rank	5	2	4	7	8	3	1	6
F85	Min	0.0022624	0.0106111	0.0073923	4.474E−07	0.0003897	0.0023305	0.0039062	2.758E−05
	Mean	0.005889	0.0217699	0.0216776	9.79E−06	0.0043743	0.0055503	0.0106483	0.000111
	Max	0.0106392	0.0355818	0.0513195	2.812E−05	0.0194865	0.0113343	0.0235451	0.000281
	Std. Dev	0.0018497	0.0059137	0.0078302	6.205E−06	0.0040837	0.0021194	0.0037311	5.179E−05
	NFEs	150,666.74	150,000	150,000	150,000	149,950	150,000	150,000	150,000
	Rank	5	8	7	1	3	4	6	2
F86	Min	2.6731641	7.5802419	34.593207	74.477091	57.675446	1.212751	5.462842	1.123634
	Mean	3.8231773	12.04304	52.078832	94.823548	68.552225	5.927061	25.284379	6.0623074
	Max	5.0428649	19.822675	82.149488	126.82104	96.23427	28.099066	52.787886	17.776648
	Std. Dev	0.5810164	2.0026695	11.591696	9.021576	7.2280658	4.94532	12.008273	3.7341928
	NFEs	150,681.24	150,000	150,000	150,000	149,950	150,000	150,000	150,000
	Rank	1	4	6	8	7	2	5	3
F87	Min	30.385334	18.526352	0.0001122	28.723718	26.525278	25.194342	24.379185	17.007979
	Mean	37.845624	24.838249	28.643671	28.830096	27.692608	149.15298	45.257047	26.657807
	Max	122.74307	28.399297	96.736648	28.97778	28.874013	1618.7818	152.59015	28.75041
	Std. Dev	22.730128	1.8644743	34.142109	0.0885231	0.567223	270.90577	33.987686	2.8855651
	NFEs	150,684.92	150,000	150,000	150,000	149,950	150,000	150,000	150,000
	Rank	6	1	4	5	3	8	7	2
F88	Min	0.1998733	0.5999739	0.1998772	0.0998733	0.0998733	0.2998734	0.3998733	0.0998733
	Mean	0.2098878	0.8757759	0.3062673	0.0998734	0.1220494	0.4598734	0.5118782	0.0998734
	Max	0.2998737	1.2001072	0.4998735	0.0998736	0.199876	0.5998734	0.6998733	0.0998736
	Std. Dev	0.0299953	0.1224258	0.0571839	3.87E−08	0.0413495	0.072111	0.0738637	5.448E−08
	NFEs	150,677.48	150,000	150,000	150,000	149,950	150,000	150,000	150,000
	Rank	4	8	5	2	3	6	7	1
F89	Min	6.5736979	0.0005756	3.114E−06	0	0	0.2894817	1.903E−06	0
	Mean	8.6250994	0.0012113	3.107E−05	0	5.979E−10	0.5957116	7.014E−06	0
	Max	11.491983	0.0026968	0.0001045	0	2.32E−08	1.1139158	1.889E−05	0
	Std. Dev	1.1481738	0.0005259	2.302E−05	0	3.261E−09	0.1792159	3.901E−06	0
	NFEs	150,613.84	150,000	150,000	25,877	128,815	150,000	150,000	6128
	Rank	8	6	5	1.5	3	7	4	1.5
F90	Min	0.0085431	4.642E−10	0	0	0	1.288E−05	0	0
	Mean	0.0157465	1.796E−07	0	0	1.511E−12	5.917E−05	1.242E−13	0
	Max	0.0236526	5.262E−06	0	0	3.997E−11	0.0001636	3.185E−12	0
	Std. Dev	0.0040431	7.512E−07	0	0	6.428E−12	3.048E−05	6.086E−13	0
	NFEs	150,676.7	150,000	108,778	19,811	97,822	150,000	129,731	3328
	Rank	8	6	2	2	5	7	4	2
F91	Min	0.0828701	2.831E−12	0	0	0	0.0001011	0	0
	Mean	0.1522853	4.172E−11	0	0	0	0.0004881	0	0
	Max	0.2357805	2.258E−10	0	0	0	0.0013272	0	0
	Std. Dev	0.0323919	4.17E−11	0	0	0	0.0002692	0	0
	NFEs	150,602.58	150,000	85,592	20,403	74,698	150,000	117,817	3283
	Rank	8	6	3	3	3	7	3	3
F92	Min	3.2490714	49.758983	11,550.227	0	0.0088312	0.3265632	0.0399548	0
	Mean	8.5558734	89.187706	22,067.937	0	242.34789	1.9688322	0.2086749	0
	Max	28.6971	144.50595	37,278.026	0	3015.2166	4.0043882	0.6984432	0
	Std. Dev	3.9890944	24.490645	5597.7904	0	548.06092	0.8114925	0.1505049	0
	NFEs	150,706.66	150,000	150,000	26,396	149,950	150,000	150,000	11,595
	Rank	5	6	8	1.5	7	4	3	1.5
F93	Min	0.0038885	4.381E−07	0	7.9598187	25.110884	0.000471	2.399E−07	0.2842164
	Mean	0.00518	1.647E−06	0.0376737	11.095765	27.02567	0.0013931	4.267E−06	2.2364866
	Max	0.0067071	4.7E−06	1.8836847	15.008003	28.578569	0.0028246	3.814E−05	6.4546824
	Std. Dev	0.0005287	9.06E−07	0.2637159	1.7561447	0.8592409	0.0005281	7.997E−06	1.4572664
	NFEs	150,652.86	150,000	101,966	150,000	149,950	150,000	150,000	150,000
	Rank	4	1	5	7	8	3	2	6
F94	Min	2.3391885	0.0167398	4.339E−08	0	0	0.5513295	1.6064746	0
	Mean	2.6786618	0.0375221	9.276E−08	0	0	1.024105	9.783841	0
	Max	2.9751368	0.0713853	1.859E−07	0	0	2.8337048	26.234636	0
	Std. Dev	0.150599	0.0104621	3.508E−08	0	0	0.4126308	6.2470217	0
	NFEs	150,623.62	150,000	150,000	32,756	82,485	150,000	150,000	8925
	Rank	7	5	4	2	2	6	8	2
F95	Min	0.2117237	1.0618729	0.0426349	0	0.0005498	0.1054729	0.1053016	0
	Mean	0.2894195	2.0829171	0.1049573	0	1.3390724	0.2105898	0.8450623	0
	Max	0.3467119	3.5026403	0.2838106	0	10.56904	0.4675521	2.9484023	0
	Std. Dev	0.0284051	0.5098974	0.0529517	0	2.2707916	0.0723034	0.6218968	0
	NFEs	150,662.86	150,000	150,000	32,129	149,950	150,000	150,000	11,203
	Rank	5	8	3	1.5	7	4	6	1.5
F96	Min	2.0722718	319.84279	3.532E−05	0	0	498.05908	5.8456756	0
	Mean	2.6263329	6.626E + 09	67.81873	0	0	1.241E + 14	127.70072	0
	Max	2.9543237	2.688E + 11	2346.6554	0	0	2.976E + 15	243.33086	0
	Std. Dev	0.164081	3.821E + 10	336.57779	0	0	5.319E + 14	76.290843	0
	NFEs	150,587.84	150,000	150,000	34,685	81,137	150,000	150,000	8965
	Rank	4	7	5	2	2	8	6	2
F97	Min	0	0	0	0	0	0	0	0
	Mean	0	0	0	0	1.013E−09	0	0	0
	Max	0	0	0	0	3.663E−08	0	0	0
	Std. Dev	0	0	0	0	5.451E−09	0	0	0
	NFEs	103,417.96	110,790	70,891	13,264	90,850	146,683	47,408	1568
	Rank	4	4	4	4	8	4	4	4
F98	Min	− 351.09111	− 315.90328	− 417.35904	− 335.79433	− 176.3927	− 326.09835	− 281.79119	− 417.10837
	Mean	− 312.26511	− 300.11098	− 243.21344	− 262.25003	− 141.70724	− 275.15033	− 237.95215	− 405.5676
	Max	− 267.34269	− 283.92668	− 166.45562	− 183.70124	− 123.84073	− 216.72155	− 199.64106	− 386.0422
	Std. Dev	19.607015	7.8121935	63.476851	28.528918	8.6292698	23.283784	23.54223	6.3389761
	NFEs	150,728.8	150,000	150,000	150,000	149,950	150,000	150,000	150,000
	Rank	2	3	6	5	8	4	7	1
F99	Min	− 186.7309	− 186.7309	− 186.73051	− 186.72297	− 186.73075	− 186.7309	− 186.7309	− 186.7309
	Mean	− 186.73085	− 186.7309	− 186.61038	− 186.22443	− 186.68061	− 186.73089	− 186.7309	− 186.72988
	Max	− 186.73052	− 186.7309	− 186.0333	− 184.33148	− 186.45089	− 186.73086	− 186.7309	− 186.72152
	Std. Dev	6.908E−05	1.421E−13	0.1415174	0.5253541	0.0608498	9.734E−06	1.421E−13	0.0017373
	NFEs	148,743.26	20,080	150,000	150,000	149,950	148,084	7756	131,832
	Rank	4	1.5	7	8	6	3	1.5	5
F100	Min	− 29.6759	− 29.6759	− 29.675666	− 29.67575	− 29.675871	− 29.6759	− 29.6759	− 29.6759
	Mean	− 29.675895	− 29.6759	− 29.657811	− 29.654279	− 29.670218	− 29.675899	− 29.6759	− 29.675841
	Max	− 29.675883	− 29.6759	− 29.593144	− 29.556596	− 29.611869	− 29.675896	− 29.6759	− 29.67492
	Std. Dev	4.212E−06	2.487E−14	0.020901	0.0227367	0.009789	8.864E−07	2.487E−14	0.0001468
	NFEs	149,466.18	25,198	150,000	150,000	149,950	149,997	8162	131,527
	Rank	4	1.5	7	8	6	3	1.5	5
F101	Min	− 25.741771	− 25.741771	− 25.741643	− 25.741739	− 25.741763	− 25.741771	− 25.741771	− 25.741771
	Mean	− 25.741767	− 25.741771	− 25.717766	− 25.736839	− 25.731797	− 25.74177	− 25.741771	− 25.741709
	Max	− 25.741747	− 25.741771	− 25.600663	− 25.703	− 25.68527	− 25.741767	− 25.741771	− 25.740868
	Std. Dev	4.117E−06	7.105E−15	0.0321383	0.00803	0.0119928	7.87E−07	7.105E−15	0.0001496
	NFEs	150,581.42	65,730	150,000	150,000	149,950	150,000	14,551	138,749
	Rank	4	1.5	8	6	7	3	1.5	5
F102	Min	8.1198442	10.364853	11.564461	0	2.0922015	9.5414678	3.2110559	1.2864735
	Mean	9.0401124	11.079985	12.210616	1.2072064	7.0504204	10.793357	5.5610738	3.3161353
	Max	10.119105	11.665728	12.63911	7.4895577	10.140474	11.718235	7.9989358	5.0044133
	Std. Dev	0.4661416	0.3066065	0.2442096	2.489496	1.8785652	0.5433896	1.0898252	0.9291788
	NFEs	150,802.54	150,000	150,000	108,193	149,950	150,000	150,000	150,000
	Rank	5	7	8	1	4	6	3	2
F103	Min	0.2440617	1.62E−06	0	0	0	0.0098693	6.999E−09	0
	Mean	0.3690504	5.496E−06	0	0	0	0.0190147	4.328E−08	0
	Max	0.4847939	1.398E−05	0	0	0	0.0344797	1.065E−07	0
	Std. Dev	0.0576115	2.724E−06	0	0	0	0.0053539	2.341E−08	0
	NFEs	150,633.72	150,000	132,933	24,700	86,320	150,000	150,000	5145
	Rank	8	6	2.5	2.5	2.5	7	5	2.5
F104	Min	0	0	0	0	0	0	0	0
	Mean	0	0	0	0	0	0.98	0.1	0
	Max	0	0	0	0	0	6	2	0
	Std. Dev	0	0	0	0	0	1.3926952	0.4123106	0
	NFEs	73,690.22	84,046	31,439	10,866	55,922	146,638	90,522	1240
	Rank	3.5	3.5	3.5	3.5	3.5	8	7	3.5
F105	Min	0.2826588	1.877E−06	2.044854	4.1248448	2.8815577	0.0092474	4.955E−09	0.0001645
	Mean	0.3854357	5.137E−06	2.7432517	6.3494812	3.7775958	0.0203817	3.614E−08	0.4792333
	Max	0.4825408	1.004E−05	3.6073175	6.7917822	4.4252885	0.0336237	9.205E−08	1.3467063
	Std. Dev	0.0523391	2.224E−06	0.3231843	0.3977501	0.2879924	0.0053202	1.965E−08	0.3514871
	NFEs	150,677.46	150,000	150,000	150,000	149,950	150,000	150,000	150,000
	Rank	4	2	6	8	7	3	1	5
F106	Min	0	0	0	0	0	0	0	0
	Mean	0	0	0	0	0	0	0	0
	Max	0	0	0	0	0	0	0	0
	Std. Dev	0	0	0	0	0	0	0	0
	NFEs	66,845.72	60,004	31,970	10,792	63,256	143,090	22,160	1264
	Rank	4.5	4.5	4.5	4.5	4.5	4.5	4.5	4.5
F107	Min	− 155	− 155	− 145	− 155	− 127	− 153	− 101	− 155
	Mean	− 155	− 154.34	− 137.1	− 155	− 113.68	− 148.16	− 75.82	− 154.36
	Max	− 155	− 152	− 130	− 155	− 103	− 139	− 59	− 150
	Std. Dev	0	0.8151074	2.8017851	0	4.7431635	2.8660775	9.5638695	1.0537552
	NFEs	9939.34	132,770	150,000	17,059	149,950	150,000	150,000	83,348
	Rank	1.5	4	6	1.5	7	5	8	3
F108	Min	4.5392844	33.642812	0.5871564	0	0	11.049074	18.117263	0
	Mean	7.4423342	41.719893	1.8513384	0	2.173E−09	29.050608	24.930286	0
	Max	21.067286	49.994433	7.1144969	0	4.058E−08	53.307162	29.591488	0
	Std. Dev	4.2316886	4.2837502	1.4147333	0	7.121E−09	9.826126	2.7641032	0
	NFEs	150,558.32	150,000	150,000	46,005	149,243	150,000	150,000	17,168
	Rank	5	8	4	1.5	3	7	6	1.5
F109	Min	0.0394756	1.428E−07	0	0	0	0.0026946	6.16E−05	0
	Mean	0.0527372	7.119E−07	0	0	0	0.0161522	0.0132389	0
	Max	0.0709243	3.6E−06	0	0	0	0.0781991	0.1184532	0
	Std. Dev	0.0074115	5.121E−07	0	0	0	0.0162108	0.0214838	0
	NFEs	150,655.6	150,000	126,098	23,981	84,764	150,000	150,000	4899
	Rank	8	5	2.5	2.5	2.5	7	6	2.5
F110	Min	− 1132.5565	− 1144.3311	− 771.06973	− 1136.6987	− 760.2832	− 1104.3007	− 1061.8912	− 1174.9832
	Mean	− 1051.9793	− 1091.4401	− 698.73085	− 1077.0015	− 647.85495	− 1024.004	− 1012.13	− 1172.6442
	Max	− 906.37007	− 1060.8122	− 644.77548	− 1031.9777	− 582.11401	− 934.65983	− 920.52403	− 1165.9043
	Std. Dev	40.407428	15.334159	25.434318	23.698519	39.821583	40.524819	37.427898	2.2004236
	NFEs	150,639.74	150,000	150,000	150,000	149,950	150,000	150,000	150,000
	Rank	4	2	7	3	8	5	6	1
F111	Min	2469.7051	0	0	0	0	2306.905	216.57404	0
	Mean	4196.5525	1233.3595	204.07436	0	0	6131.6934	1266.4692	0
	Max	4889.7299	5963.5903	3651.9685	0	0	12,036.516	2429.471	0
	Std. Dev	518.27442	1433.9371	570.3299	0	0	2394.5309	446.05259	0
	NFEs	150,547.04	142,194	113,762	39,498	1050	150,000	150,000	3340
	Rank	7	5	4	2	2	8	6	2
F112	Min	164.60841	71.949683	137.78151	24.696335	24.468051	147.67746	89.757421	24.660514
	Mean	186.51224	99.815878	164.06475	24.933105	51.736085	193.73563	149.36768	47.970949
	Max	200.83196	119.10462	198.38504	25.49441	125.9227	296.34431	217.15848	81.606885
	Std. Dev	8.3658057	10.267658	12.871777	0.1797349	29.714679	29.640256	33.48754	14.230126
	NFEs	150,683.78	150,000	150,000	150,000	149,950	150,000	150,000	150,000
	Rank	7	4	6	1	3	8	5	2
F113	Min	3.53E−10	0	0	0	0	1.266E−10	0	0
	Mean	3.232E−08	0	0	0	0	9.353E−09	0	0
	Max	1.135E−07	0	0	0	0	6.292E−08	0	0
	Std. Dev	2.778E−08	0	0	0	0	1.189E−08	0	0
	NFEs	150,888.56	32,514	19,074	18,720	5033	150,000	9700	1203
	Rank	8	3.5	3.5	3.5	3.5	7	3.5	3.5
F114	Min	1.2169811	2.8947126	0.0003839	0	0	2.6871831	5.3315469	0
	Mean	2.1666108	4.9259417	0.5153793	0	0	6.8368507	10.175872	0
	Max	4.3834416	6.6516057	3.0006156	0	0	12.269068	15.287907	0
	Std. Dev	1.051405	0.8596602	0.7666543	0	0	2.370051	2.0501896	0
	NFEs	150,626.5	150,000	150,000	31,220	75,108	150,000	150,000	7998
	Rank	5	6	4	2	2	7	8	2
F115	Min	400.22651	454.44163	0	391.02803	389.03203	433.67796	562.77178	302.06433
	Mean	423.99483	524.24823	351.00674	397.08273	412.30851	577.26351	655.25655	369.01597
	Max	642.81689	572.63484	780.45785	407.5249	561.32999	708.68331	749.79498	399.74704
	Std. Dev	36.734968	27.542317	342.59666	4.3539142	31.883607	67.316754	48.127237	17.662407
	NFEs	150,739.18	150,000	127,943	150,000	149,950	150,000	150,000	150,000
	Rank	5	6	1	3	4	7	8	2
F116	Min	1.946E−06	7.383E−05	1.662E−09	0	0	7.381E−06	3.336E−06	0
	Mean	0.0088639	0.0018742	0.1221956	0	3.437E−07	0.1088226	0.0003463	0
	Max	0.2195172	0.0077134	3.0643003	0	1.248E−05	1.7435142	0.0035676	0
	Std. Dev	0.0334188	0.001761	0.4930401	0	1.774E−06	0.3030003	0.0007079	0
	NFEs	150,616.42	150,000	150,000	52,287	108,203	150,000	150,000	3222
	Rank	6	5	8	1.5	3	7	4	1.5
F117	Min	8.443E−12	1.136E−11	2.747E−11	4.069E−12	4.788E−11	7.036E−12	3.512E−12	3.819E−12
	Mean	1.204E−11	1.742E−11	2.973E−11	4.929E−12	2.436E−10	1.187E−11	3.662E−12	5.446E−12
	Max	1.718E−11	2.082E−11	3.157E−11	5.921E−12	7.245E−10	2.39E−11	4.273E−12	8.028E−12
	Std. Dev	1.929E−12	2.134E−12	7.558E−13	4.596E−13	1.307E−10	3.605E−12	1.845E−13	9.152E−13
	NFEs	150,758.46	150,000	150,000	150,000	149,950	150,000	150,000	150,000
	Rank	5	6	7	2	8	4	1	3
F118	Min	7.79E−232	4.34E−232	4.34E−232	5.4E−222	9.02E−217	7.52E−224	4.54E−114	4.34E−232
	Mean	1.33E−231	4.34E−232	6.32E−229	2.12E−209	6.04E−197	7.79E−178	7.19E−70	4.34E−232
	Max	1.74E−231	4.34E−232	2.39E−227	1.05E−207	1.12E−195	3.88E−176	3.137E−68	4.34E−232
	Std. Dev	0	0	0	0	0	0	4.393E−69	0
	NFEs	150,615.08	150,000	150,000	150,000	149,950	150,000	150,000	150,000
	Rank	3	1.5	4	5	6	7	8	1.5
F119	Min	2.818E−12	2.983E−12	2.473E−09	2.979E−12	1.117E−09	2.808E−12	2.807E−12	2.814E−12
	Mean	3.191E−12	3.061E−12	8.026E−09	3.25E−12	5.225E−09	6.38E−12	2.862E−12	2.882E−12
	Max	6.056E−12	3.158E−12	1.9E−08	3.669E−12	1.857E−08	2.162E−11	5.565E−12	3.04E−12
	Std. Dev	9.187E−13	4.489E−14	3.817E−09	1.757E−13	3.202E−09	5.311E−12	3.861E−13	5.743E−14
	NFEs	150,664.42	150,000	150,000	150,000	149,950	150,000	150,000	150,000
	Rank	4	3	8	5	7	6	1	2
F120	Min	0.0118379	8.6378582	46.641399	0	3.576E−06	0.001705	2.5883177	0
	Mean	0.0221057	13.359538	75.00896	0	0.0855598	0.0038338	6.3506408	0
	Max	0.0539693	19.713932	106.77373	0	2.5400129	0.0068168	11.903133	0
	Std. Dev	0.0080628	2.6657201	14.445587	0	0.358683	0.001097	2.4237925	0
	NFEs	150,598.88	150,000	150,000	27,196	149,950	150,000	150,000	17,673
	Rank	4	7	8	1.5	5	3	6	1.5

### Non-parametric statistical analyses

Non-parametric statistical methods are useful tools for comparing and ranking the performance of metaheuristic algorithms. In this study, four well-known non-parametric tests including the Wilcoxon Signed-Rank^98^, Friedman^99^, Friedman Aligned Ranks^100^, and Quade^101^ tests, are used to analyze the ability of algorithms in solving benchmark problems; in all of these tests, the significance level, $\alpha$, is 0.05^102^.

The results of the Wilcoxon Signed-Rank test are presented in Table 7, which shows that the *R*^+^ of FuFiO is less than the *R*^−^ of all the other methods, which means that FuFiO performs better than all of the compared ones. Furthermore, the *p*-values show that the FuFiO algorithm significantly outperforms other algorithms in solving benchmark problems, except in competition with the CS and CSA algorithms in solving the fixed-dimensional problems.

**Table 7:** The Wilcoxon Signed-Rank test results.

One-to-one comparison	Type	*R*^+^	*R*^-^	*T*	*p*-value
FuFiO vs. FA	Fixed-dimensional	0	1596	0	7.5475E−11
	*N*-dimensional	269	1327	269	1.5953E−05
FuFiO vs. CS	Fixed-dimensional	2	13	2	0.13801074
	*N*-dimensional	273	1158	273	8.9528E−05
FuFiO vs. Jaya	Fixed-dimensional	0	406	0	3.7896E−06
	*N*-dimensional	182	899	182	8.9752E−05
FuFiO vs. TEO	Fixed-dimensional	0	820	0	3.5694E−08
	*N*-dimensional	115	381	115	0.00915154
FuFiO vs. SCA	Fixed-dimensional	0	780	0	5.2553E−08
	*N*-dimensional	29	961	29	5.3788E−08
FuFiO vs. MVO	Fixed-dimensional	0	1653	0	5.1438E−11
	*N*-dimensional	248	1405	248	4.3005E−06
FuFiO vs. CSA	Fixed-dimensional	7	21	7	0.23672357
	*N*-dimensional	199	1232	199	4.8205E−06

The Friedman test is a ranking method the results of which are presented in Table 8. According to this test, the FuFiO algorithm is placed in the first rank in all types of problems.

**Table 8:** The Friedman test results.

Method	Type			
	Fixed-dimensional		*N*-dimensional	
	*R*	Rank	*R*	Rank
FA	6.1916667	8	5.0833333	7
CS	2.775	2	4.5583333	3
Jaya	4.7166667	4	4.9583333	6
TEO	5.875	7	3.525	2
SCA	5.35	5	4.9333333	5
MVO	5.6833333	6	5.5083333	8
CSA	2.8083333	3	4.65	4
FuFiO	2.6	1	2.7833333	1
Statistic	163.69444		56.783333	
*p*-value	5.351E−32		6.601E−10	

In the Friedman Aligned Rank test, the average of each set of values is calculated and then subtracted from the results. Subsequently, this method ranks algorithms based on their corresponding shifted values which are called aligned ranks. The results of this test, presented in Table 9, show that the FuFiO algorithm gains the first rank in solving both fixed- and *N*-dimensional benchmark problems.

**Table 9:** The Friedman aligned ranks test results.

Method	Type			
	Fixed-dimensional		*N*-dimensional	
	*R*	Rank	*R*	Rank
FA	259.6917	6	232.4167	3
CS	194.7917	3	240.7167	4
Jaya	250	5	270.7083	7
TEO	328.5583	8	225.7583	2
SCA	230.1833	4	266.6333	6
MVO	279.3667	7	276.7417	8
CSA	194.2083	2	241.9333	5
FuFiO	187.2	1	169.0917	1
Statistic	73.96375		29.77014	
*p*-value	2.33E−13		0.000105	

The Quade test can be considered as an extension of the Wilcoxon Signed-Rank test for comparing multiple algorithms, making it often more effective than the previous tests. The results of the Quade test are presented in Table 10, showing that the FuFiO method is ranked first in comparison with the other methods for all types of problems.

**Table 10:** The Quade test results.

Method	Type			
	Fixed-dimensional		*N*-dimensional	
	*R*	Rank	*R*	Rank
FA	5.870219	7	4.813661	4
CS	2.753552	3	4.70082	3
Jaya	4.843989	4	4.903279	5
TEO	6.26612	8	3.521858	2
SCA	5.572678	6	4.990984	6
MVO	5.543716	5	5.520219	8
CSA	2.709016	2	5.048361	7
FuFiO	2.44071	1	2.50082	1
Statistic	27.94946		8.282949	
*p*-value	0.000225		0.308306	

**Figure 13:**
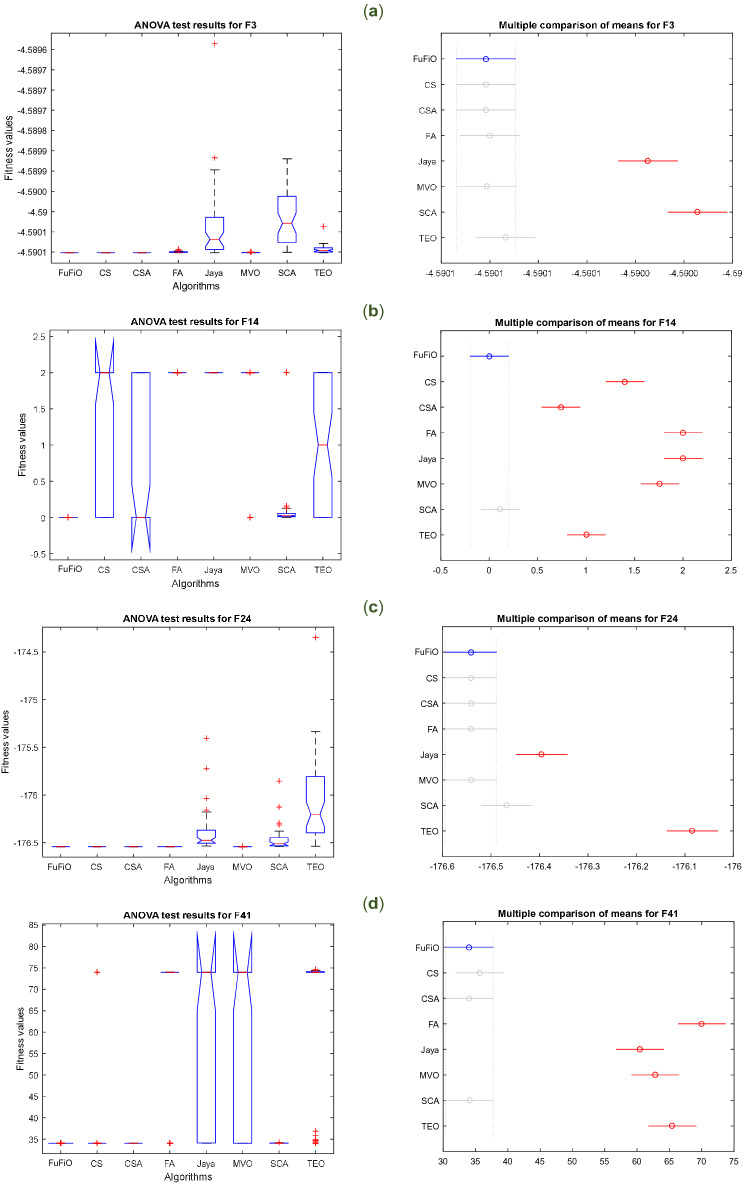
ANOVA test results for fixed-dimension functions.

**Figure 14:**
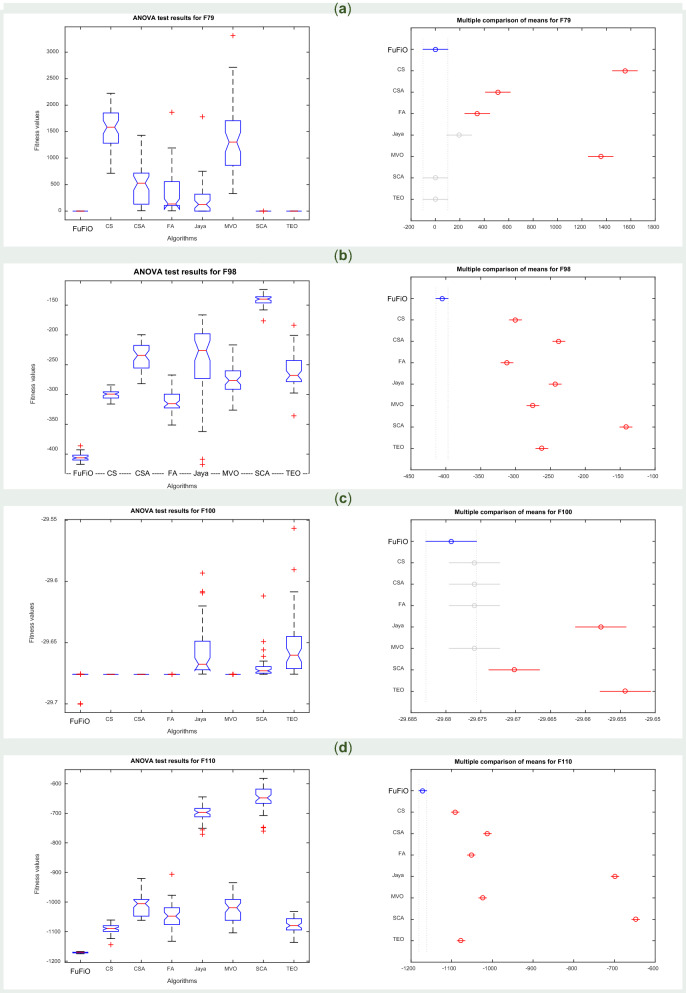
ANOVA test results for *N*-dimension functions.

The final statistical method considered here is the analysis of variance (ANOVA) test, which compares the variance of results across the means of various algorithms. In this research, the ANOVA test has been employed with a significance level of 5% to study the efficiency and relative performance of optimizers. The results of this test are presented in Table 11. According to these results, the *p*-values indicate significant differences between the means in the majority of the considered problems. Besides, the results of the ANOVA test for four fixed-dimension and four *N*-dimension problems are plotted in Figs. 13 and 14, respectively.

**Table 11:** Results of the ANOVA test.

No	*F*	*p-value*	No	*F*	*p-value*	No	*F*	*p-value*
1	212.628	3E−129	41	91.4162	2.1E−78	81	163.779	3E−112
2	18.3774	3.6E−21	42	60.4215	1.6E−58	82	138.653	6E−102
3	38.6995	3.4E−41	43	42.0432	4.3E−44	83	984.778	3E−244
4	9.41856	8.2E−11	44	32.5073	1.5E−35	84	843.09	7E−232
5	95.5752	9.2E−81	45	23.1121	3.1E−26	85	219.699	2E−131
6	55.7522	4.5E−55	46	14.172	2E−16	86	1016.25	7E−247
7	26.3995	1.3E−29	47	41.745	7.7E−44	87	9.15264	1.7E−10
8	51.0346	1.9E−51	48	4.78228	3.5E−05	88	909.013	7E−238
9	44.5832	3.1E−46	49	7.9035	5.6E−09	89	2658.81	0
10	11.2979	4.6E−13	50	59.8849	4E−58	90	742.438	9E−222
11	0.97232	0.45099	51	137.807	1E−101	91	1081.97	6E−252
12	20.3401	2.6E−23	52	158.415	4E−110	92	751.033	1E−222
13	67.4868	1.8E−63	53	25.6152	8.1E−29	93	6092.99	0
14	75.3191	1.2E−68	54	44.9057	1.7E−46	94	115.783	2.4E−91
15	21.5699	1.3E−24	55	38.4801	5.4E−41	95	38.7123	3.3E−41
16	54.0947	8.2E−54	56	5.43352	5.8E−06	96	2.6681	0.01044
17	14.3731	1.2E−16	57	62.3563	6.7E−60	97	1.69325	0.10904
18	19.2364	4.2E−22	58	53.5171	2.3E−53	98	338.736	7E−162
19	24.8055	5.4E−28	59	18.3172	4.2E−21	99	40.3648	1.2E−42
20	18.5329	2.4E−21	60	621.352	9E−208	100	30.853	5.6E−34
21	18.6236	1.9E−21	61	119.601	3.3E−93	101	19.0169	7.2E−22
22	25.0435	3.1E−28	62	73.4034	2E−67	102	491.788	1E−189
23	32.6928	1E−35	63	2997.81	0	103	1969.43	6E−301
24	41.7835	7.2E−44	64	1143.49	2E−256	104	21.8893	5.9E−25
25	38.3024	7.7E−41	65	352.692	7E−165	105	4608.94	0
26	67.8982	9.4E−64	66	NaN	NaN	106	NaN	NaN
27	53.9871	9.9E−54	67	1461.31	2E−276	107	2405.03	0
28	42.3063	2.6E−44	68	2258.09	0	108	741.745	1E−221
29	139.703	2E−102	69	16.6731	2.8E−19	109	170.616	8E−115
30	3.12764	0.00317	70	5645.22	0	110	1855.05	5E−296
31	3.12116	0.00323	71	13.0153	4.4E−15	111	242.365	4E−138
32	7.10771	5.3E−08	72	602.852	2E−205	112	518.713	8E−194
33	16.5702	3.7E−19	73	41.1597	2.5E−43	113	56.1407	2.3E−55
34	23.754	6.6E−27	74	2.84673	0.0066	114	474.154	6E−187
35	7.57138	1.4E−08	75	8.7876	4.8E−10	115	36.6578	2.3E−39
36	52.7857	8.2E−53	76	3.91161	0.00039	116	3.25476	0.00227
37	1.76362	0.09318	77	88.268	1.4E−76	117	155.142	8E−109
38	15.0353	2E−17	78	18.4005	3.4E−21	118	1.31254	0.2428
39	1.71051	0.10494	79	171.251	5E−115	119	157.206	1E−109
40	10.0222	1.5E−11	80	36.5495	2.8E−39	120	1192.05	9E−260

*NaN means there is no difference between means.

## Analyses based on competitions on evolutionary computation (CEC)

In this section, the performance of the FuFiO algorithm is investigated using the single-objective real-parameter numerical optimization problems of two recent Competitions on Evolutionary Computation, namely CEC-2017 and CEC-2019 benchmark test functions. Then, the computational time and complexity of FuFiO is compared with other state-of-the-art algorithms.

### Comparative analyses based on the CEC-2017 test functions

To investigate the ability of FuFiO in solving more difficult problems, the CEC 2017 Special Season on single-objective problems are utilized in this sub-section. To establish and perform a comparative analysis, four state-of-the-art algorithms including the Effective Butterfly Optimizer with Covariance Matrix Adapted Retreat (EBOwithCMAR)^103^, ensemble sinusoidal differential covariance matrix adaptation with Euclidean neighborhood (LSHADE-cnEpSin)^104^, Multi-Method-based Orthogonal Experimental Design (MM_OED)^105^], and Teaching Learning Based Optimization with Focused Learning (TLBO-FL)^106^ are considered. Table 12 contains a list of these problems the mathematical details of which was presented by the CEC 2017 committee^107^.

**Table 12:** Summary of the CEC-2017 test functions.

No	Function	*D*	Min
C1	Shifted and Rotated Bent Cigar Function	10, 30, 50, 100	0
C2	Removed by committee	–	–
C3	Shifted and Rotated Zakharov Function	10, 30, 50, 100	0
C4	Shifted and Rotated Rosenbrock’s Function	10, 30, 50, 100	0
C5	Shifted and Rotated Rastrigin’s Function	10, 30, 50, 100	0
C6	Shifted and Rotated Expanded Scaffer’s F6 Function	10, 30, 50, 100	0
C7	Shifted and Rotated Lunacek Bi_Rastrigin Function	10, 30, 50, 100	0
C8	Shifted and Rotated Non-Continuous Rastrigin’s Function	10, 30, 50, 100	0
C9	Shifted and Rotated Levy Function	10, 30, 50, 100	0
C10	Shifted and Rotated Schwefel’s Function	10, 30, 50, 100	0
C11	Hybrid Function 1 (*N* = 3)	10, 30, 50, 100	0
C12	Hybrid Function 2 (*N* = 3)	10, 30, 50, 100	0
C13	Hybrid Function 3 (*N* = 3)	10, 30, 50, 100	0
C14	Hybrid Function 4 (*N* = 4)	10, 30, 50, 100	0
C15	Hybrid Function 5 (*N* = 4)	10, 30, 50, 100	0
C16	Hybrid Function 6 (*N* = 4)	10, 30, 50, 100	0
C17	Hybrid Function 6 (*N* = 5)	10, 30, 50, 100	0
C18	Hybrid Function 6 (*N* = 5)	10, 30, 50, 100	0
C19	Hybrid Function 6 (*N* = 5)	10, 30, 50, 100	0
C20	Hybrid Function 6 (*N* = 6)	10, 30, 50, 100	0
C21	Composition Function 1 (*N* = 3)	10, 30, 50, 100	0
C22	Composition Function 2 (*N* = 3)	10, 30, 50, 100	0
C23	Composition Function 3 (*N* = 4)	10, 30, 50, 100	0
C24	Composition Function 4 (*N* = 4)	10, 30, 50, 100	0
C25	Composition Function 5 (*N* = 5)	10, 30, 50, 100	0
C26	Composition Function 6 (*N* = 5)	10, 30, 50, 100	0
C27	Composition Function 7 (*N* = 6)	10, 30, 50, 100	0
C28	Composition Function 8 (*N* = 6)	10, 30, 50, 100	0
C29	Composition Function 9 (*N* = 3)	10, 30, 50, 100	0
C30	Composition Function 10 (*N* = 3)	10, 30, 50, 100	0

The statistical results of FuFiO and the other algorithms in solving 10-, 30-, 50- and 100-dimensional problems are presented in Tables 13, 14, 15, and 16, respectively. These results are based on 51 independent runs. An error value is considered in this study such that when it is less than 10^−8^, the error is considered zero. The total number of function evaluations for each test problem is taken as 10000*D*, where *D* is the problem dimension. The results confirm that the FuFiO method can provide very competitive results.

**Table 13:**
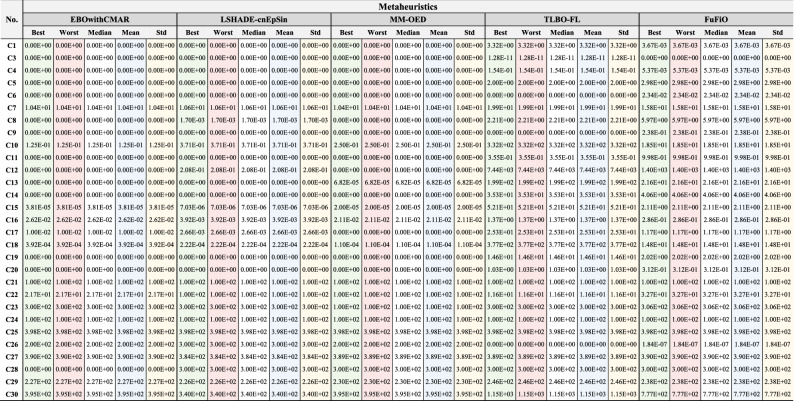
Statistical results of different algorithms for the 10-dimensional CEC-2017 problems.

**Table 14:**
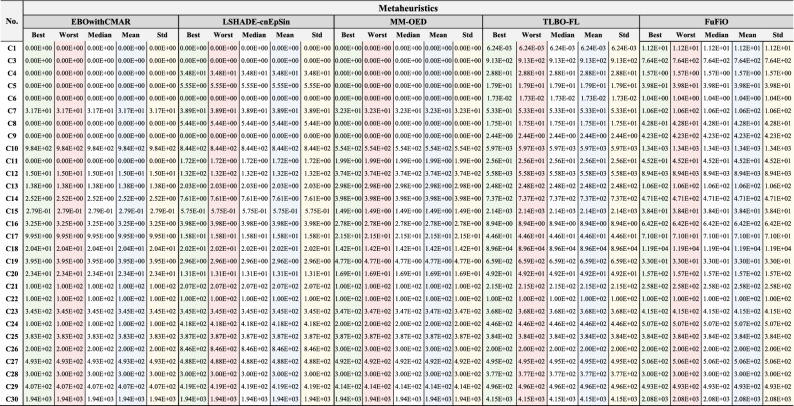
Statistical results of different algorithms for the 30-dimensional CEC-2017 problems.

**Table 15:**
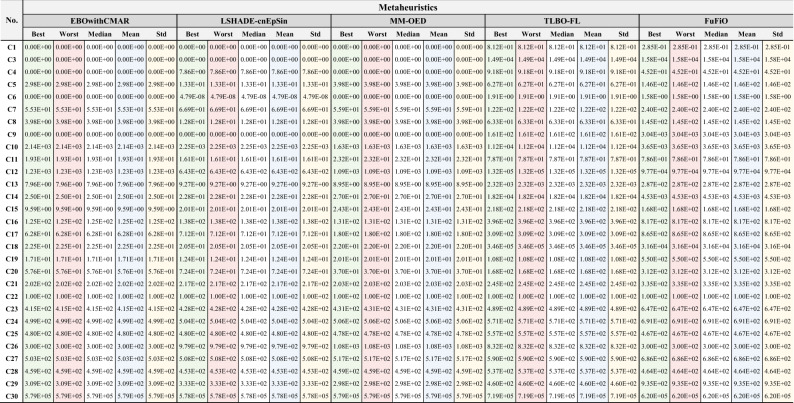
Statistical results of different algorithms for the 50-dimensional CEC-2017 problems.

**Table 16:**
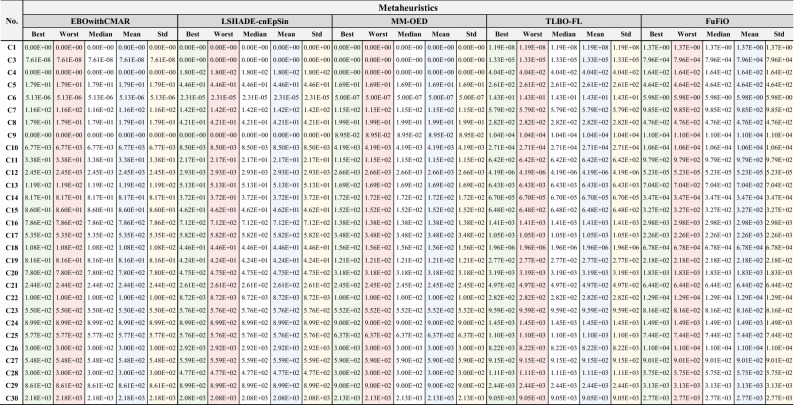
Statistical results of different algorithms for the 100-dimensional CEC-2017 problems.

### Computational time and complexity analyses

**Figure 15:**
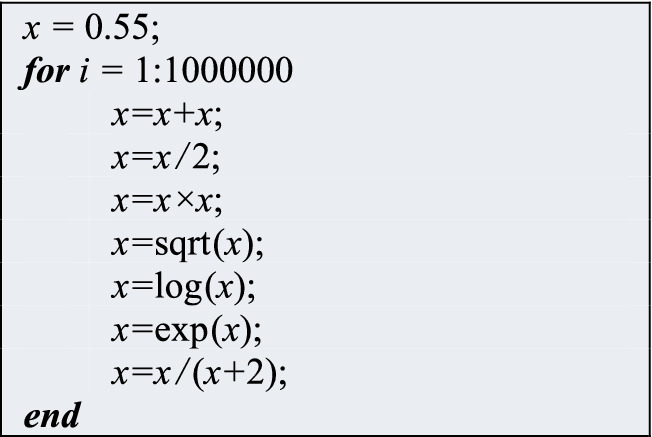
Procedure of *T*_0_ assessment.

A complete computational time and complexity analysis is conducted to evaluate the FuFiO algorithm. Awad et al. have proposed a simple procedure to analyze the complexity of metaheuristic algorithms in the CEC-2017 instructions^107^, in which complexity is reflected by four times, namely ${T}_{0}$, ${T}_{1}$, ${T}_{2}$, and $\widehat{{T}_{2}}$, as follows: ${T}_{0}$ is the computing time of the test program shown in Fig. 15; ${T}_{1}$ is given by the time of 200,000 evaluations of ${F}_{18}$ by itself with *D* dimensions; ${T}_{2}$ is the total computing time of the FuFiO algorithm in 200,000 evaluations of the same *D*-dimensional ${F}_{18}$; and $\widehat{{T}_{2}}$ denotes the mean value of five different runs of ${T}_{2}$.

The complexity results of the FuFiO algorithm and other methods in 10, 30, 50, and 100 dimensions are presented in Table 17, which demonstrate that FuFiO can perform competitively.

**Table 17:** Computational complexity of the FuFiO algorithm versus the other algorithms.

*D*	Time	EBOwithCMAR	LSHADE-cnEpSin	MM-OED	TLBO-FL	FuFiO
10	${T}_{0}$	0.0413	0.1093	2.157784	0.09	0.053148815
	${T}_{1}$	0.8218	0.8391	0.146416	0.41	0.919610921
	$\widehat{{T}_{2}}$	7.5794	2.1835	6.704923	1.62	6.692289658
	$\widehat{{T}_{2}}-{T}_{1}/{T}_{0}$	163.622276	12.30009149	3.039464098	13.44444444	108.6134986
30	${T}_{1}$	1.1507	1.057	0.592848	0.79	1.408477381
	$\widehat{{T}_{2}}$	6.591	3.6724	20.84485	2.17	8.167910826
	$\widehat{{T}_{2}}-{T}_{1}/{T}_{0}$	131.7263923	23.92863678	9.385555737	15.33333333	127.179382
50	${T}_{1}$	1.8792	1.4338	1.606688	1.45	2.256751371
	$\widehat{{T}_{2}}$	8.7886	3.7066	38.51665	3.03	9.881637315
	$\widehat{{T}_{2}}-{T}_{1}/{T}_{0}$	167.2978208	20.79414456	17.10549434	17.55555556	143.462953
100	${T}_{1}$	5.6887	3.0237	5.776893	4.81	6.769188826
	$\widehat{{T}_{2}}$	18.4969	7.7564	72.62159	6.93	16.64127159
	$\widehat{{T}_{2}}-{T}_{1}/{T}_{0}$	310.125908	43.30009149	30.97840053	23.55555556	185.7441744

The key metric in evaluating the running time of an algorithm is computational complexity, which is defined based on its structure. According to Big O notation, the complexity of the FuFiO algorithm is calculated based on the number of nuclei *n*, number of design variables *d*, maximum number of iterations *t*, and the sorting mechanism of nuclei in each iteration as follows:

$$ \begin{aligned}O\left(FuFiO\right)&=O\left(t\times \left[O\left(sort\right)+O\left(nuclear\, reaction\, level\right)\right]\right)\\&=O\left(t\times \left[{n}^{2}+n\times d\right]\right)\\&=O(t{n}^{2}+nd)\end{aligned} $$

### Comparative analyses based on the CEC-2019 test functions

In this sub-section, the problems defined by the CEC-2019 Special Season are utilized. Different physics-based methods including the Gravitational Search Algorithm (GSA)^86^ and Electromagnetic Field Optimization (EFO)^56^. Furthermore, three recently-developed evolutionary methods including the Farmland Fertility Algorithm (FFA)^35^, African Vultures Optimization Algorithm (AVOA)^37^, and Artificial Gorilla Troops Optimizer (GTO)^42^, are considered for this comparative study. Table 18 presents the properties of the CEC-2019 examples^108^.

**Table 18:** Summary of the CEC 2019 test functions.

No	Function	*D*	Limits
C1	Storn's Chebyshev Polynomial Fitting Problem	9	[− 8192, 8192]
C2	Inverse Hilbert Matrix Problem	16	[− 16384, 16,384
C3	Lennard–Jones Minimum Energy Cluster	18	[− 4,4]
C4	Rastrigin’s Function	10	[− 100,100]
C5	Griewangk’s Function	10	[− 100,100]
C6	Weierstrass Function	10	[− 100,100]
C7	Modified Schwefel’s Function	10	[− 100,100]
C8	Expanded Schaffer’s F6 Function	10	[− 100,100]
C9	Happy Cat Function	10	[− 100,100]
C10	Ackley Function	10	[− 100,100]

**Figure 16:**
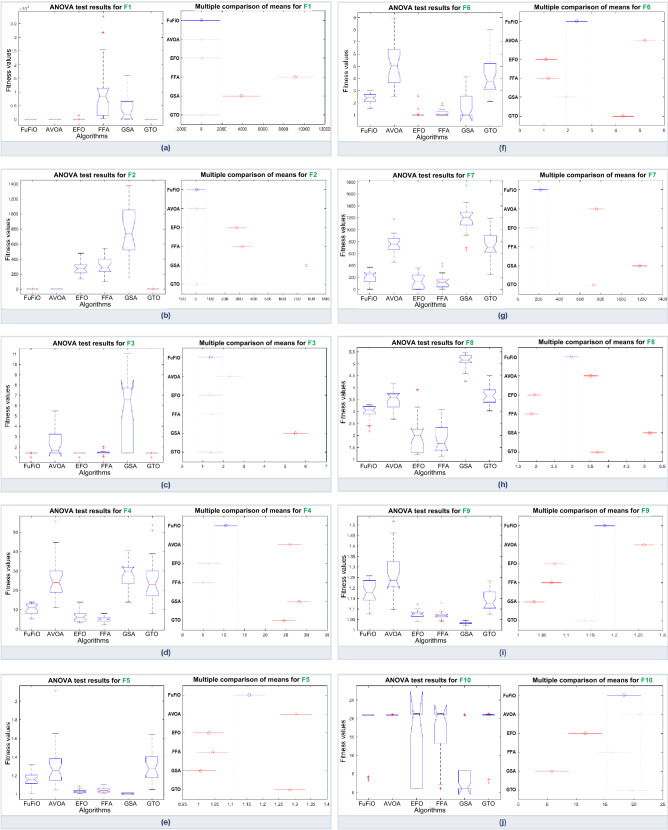
ANOVA test results for the CEC-2019 functions.

The statistical results of the algorithms are presented in Table 19. These results are based on 50 independent runs, but for reporting the final result, we select the best 25 ones according to the CEC-2019 rules. An error value is considered in this study such that when it is less than 10^−10^, the error is considered zero. The total number of function evaluations for each test problem is taken as 10^6^. A conclusion concerning the statistical results is also added to the table. The final output shows that FuFiO is placed in the second place with a very small difference while its stability in finding results is so far better that the other methods based on the standard divination values. Moreover, the ANOVA test has been employed with a significance level of 5% and the related results for all problems are plotted in Fig. 16. The results show a good performance of the present method for many of the examined functions.

**Table 19:** Statistical results of different algorithms for the CEC-2019 problems.

No	Statistics	Methods					
		AVOA	EFO	GSA	GTO	FFA	FuFiO
F1	Min	1	1.0000	8.3415	1	345.00	1
	Mean	1	56.719	3877.1	1	9118.7	1
	Max	1	1320.8	16,074.4	1	37,546.3	1
	Std. Dev	0	263.7365	4954.383	0	9680.685	0
	Rank	1	4	5	1	6	1
F2	Min	4.1582	136.50	146.73	4.3429	102.31	4.07647
	Mean	4.4786	282.26	763.01	4.3015	318.49	4.2940
	Max	5.0000	479.01	1379.8	4.2323	539.96	4.5248
	Std. Dev	0.3368	90.761	358.24	0.2222	114.97	0.1079
	Rank	3	4	6	2	5	1
F3	Min	1.4091	1	1.4091	1.4091	1.0213	1
	Mean	2.2820	1.3600	5.4944	1.3764	1.4650	1.3764
	Max	5.4761	1.4091	11.062	1.4091	2.0300	1.4091
	Std. Dev	1.2939	0.1356	3.3347	0.1132	0.23135	0.1132
	Rank	5	1	6	2	4	2
F4	Min	10.949	2.9899	13.934	29.853	2.0010	5.1018
	Mean	25.917	6.3727	28.192	24.57	5.0354	10.478
	Max	55.722	13.934	40.798	10.949	7.9884	13.934
	Std. Dev	9.8415	2.4371	6.7920	11.344	1.5161	2.6720
	Rank	5	2	6	4	1	3
F5	Min	1.0443	1.0073	1	1.2019	1.0098	1.0098
	Mean	1.3033	1.0308	1.0051	1.2848	1.0432	1.1575
	Max	2.1119	1.0787	1.0123	1.2263	1.1074	1.3149
	Std. Dev	0.2309	0.0176	0.0056	0.1598	0.0265	0.0781
	Rank	6	2	1	5	3	4
F6	Min	2.5555	1	1.0000	5.2358	1	1.5501
	Mean	5.2067	1.0913	1.9335	4.2949	1.1720	2.3619
	Max	8.9700	2.5784	4.1527	3.1026	2.0007	2.9909
	Std. Dev	1.7852	0.3270	1.1032	1.563	0.2715	0.3842
	Rank	6	1	3	5	2	4
F7	Min	456.5332	1.0624	652.48	629.81	4.6023	1.4371
	Mean	757.44	129.58	1177.514	730.64	133.6176	214.4899
	Max	1177.4	360.58	1741.3	630.92	432.81	368.39
	Std. Dev	160.64	120.70	233.21	271.69	114.88	97.056
	Rank	5	1	6	4	2	3
F8	Min	2.6710	1.2071	4.2678	3.4973	1.1316	2.1755
	Mean	3.5125	1.9530	5.1443	3.6896	1.8749	2.9748
	Max	4.1638	3.9034	5.4618	3.1992	3.0803	3.2865
	Std. Dev	0.4250	0.7031	0.2736	0.4179	0.5817	0.3140
	Rank	4	2	6	5	1	3
F9	Min	1.0977	1.0403	1.0225	1.1049	1.0410	1.0760
	Mean	1.2612	1.0754	1.0326	1.1378	1.0693	1.1797
	Max	1.5168	1.122754	1.0457	1.1782	1.1296	1.2576
	Std. Dev	0.1051	0.01727	0.0057	0.0481	0.0176	0.0524
	Rank	6	3	1	4	2	5
F10	Min	20.988	1	1.0000	21.130	1.0000	3.3168
	Mean	21.018	11.564	5.7999	19.654	16.431	18.254
	Max	21.240	21.303	21.000	21.125	21.311	21.000
	Std. Dev	0.0545	10.267	8.7176	4.9808	8.3136	6.4201
	Rank	6	2	1	5	3	4
*Total* *Rank* *Based on:*	Min	4.1	2.2	4	5	2.95	2.75
	Mean	4.8	2.2	4.1	3.9	2.9	3.1
	Max	4.8	2.85	4.2	2.8	3.3	3.05
	Std. Dev	4.2	3.4	3.8	3.7	3.4	2.5
	Total	4.475	2.6625	4.025	3.85	3.1375	2.85

## Conclusions and future work

Inspired by the concept of nuclei stability in physics, we developed a swarm-based intelligence metaheuristic method, called Fusion Fission Optimization (FuFiO), to deal with various optimization problems. In this method, three nuclear reactions including fusion, fission, and $\beta$-decay are modeled to simulate the tendency to change a stable nuclei.

The effectiveness of the FuFiO algorithm in solving optimization problems with better results can be related to its mechanism for creating the right balance between exploration and exploitation. Also, in the FuFiO method, three different reactions are proposed for each group with novel formulations. The search procedure of each reaction in each group can be interpreted as follows:

- **Fusion**: Through this reaction, a nucleus in the stable group slams with another stable nucleus and exploits the search space. On the other hand, this operator explores the search space in the unstable group because the unstable nuclei slam with each other.
- **Fission**: Through this reaction, in the first group, a stable nucleus slams with an unstable one that explores the search space around the stable nucleus. On the other hand, in the second group, the fission operator guides the unstable nuclei toward the stable region to exploit it.
- ${\varvec{\beta}}$**-decay**: According to these operators, a stable nucleus slams with a randomly-generated nucleus, which results in exploration. However, in the second group, $\beta$-decay generates the new solution by a uniform crossover between the unstable nucleus and a stable one to transfer some stable features to the unstable nucleus.

The right balance between exploration and exploitation is guaranteed by randomness in selecting the reactions in each group algorithm.

To examine the performance of FuFiO in comparison with seven well-known optimizers, an extensive set of 120 benchmark problems were considered, where the obtained results were used as the inputs of several non-parametric statistical methods. The results of statistical analysis showed that the FuFiO algorithm has a superior performance in solving all considered types of problems. To further investigate the ability of FuFiO in solving complex optimization problems, the CEC 2017 and CEC 2019 was utilized. The results showed that the FuFiO algorithm can perform competitively when compared to the state-of-the-art algorithms.

Despite the good performance of FuFiO in solving different well-studied mathematical problems, this method, like other metaheuristics, may have some limitations for solving difficult constrained or engineering problems. The main reason is the influence of the utilized constraint-handling approach on the performance of the proposed method. In addition, for more complex problems where each function evaluation needs a considerable amount of time, applying this method may need further investigations. Importantly, not the advantages of the new method, but its limitations open up a new avenue to improve or adapt it for applications in other fields.

Future studies concerning the FuFiO algorithm can be classified into two main categories. The first category contains investigations in which FuFiO is utilized as an optimization solver in dealing with complex real-world optimization problems. The second category concerns modifying the FuFiO algorithm to enhance its computational accuracy and efficiency. To this end, various kinds of modification can be designed, some of which are as follows:

1. The proposed algorithm has two parameters, namely ${U}_{s}$ and ${L}_{s}$. The value of ${U}_{s}$ is determined according to the natural ratio of stable nuclei, whereas the value of ${L}_{s}$ is decided empirically. These parameters and their effects should be studied more thoroughly.
2. In this paper, as the first version of the algorithm, the value of ${S}_{z}$ is determined through a deterministic procedure. A more advanced approach could be developed to define the size of stable nuclei.
3. For updating the position of nuclei, in each group, three different reactions are modeled. In order to enhance the performance of the algorithm, developing new formulations for reactions could be advantageous.
4. In each reaction, another stable or unstable nucleus, ${X}_{j}$, is selected randomly. Using a more thoughtful, systematic selection method could improve the performance of the algorithm.
5. During the updating process, a reaction is randomly selected without any specific rule. Developing a deterministic, adaptive, or self-adaptive approach to choosing an appropriate reaction could improve the algorithm.

In addition to the abovementioned approaches, one may use alternative strategies to improving the FuFiO algorithm. For example, as a conventional approach, the hybridization of the proposed algorithm with other popular metaheuristic algorithms could lead to the development of more robust optimization algorithms.

## Data Availability

The datasets used and/or analysed during the current study are available from the corresponding author on reasonable request.
