# Author Correction: The fusion–fission optimization (FuFiO) algorithm

**DOI:** 10.1038/s41598-022-18952-9

**Published:** 2022-09-02

**Authors:** Behnaz Nouhi, Nima Darabi, Pooya Sareh, Hadi Bayazidi, Farhad Darabi, Siamak Talatahari

**Affiliations:** 1 Department of Mathematical Sciences, University of Tabriz, Tabriz, Iran; 2 Department of Civil, Constructional and Environmental Engineering, Sapienza University of Rome, Via Eudossiana, 18, 00184 Rome, Italy; 3 Creative Design Engineering Laboratory (Cdel), Department of Mechanical, Materials, and Aerospace Engineering, School of Engineering, University of Liverpool, Liverpool, L69 3GH UK; 4 Department of Civil Engineering, University of Tabriz, Tabriz, Iran; 5 Department of Physics, Azarbaijan Shahid Madani University, Tabriz, 53714-161 Iran; 6 Faculty of Engineering and Information Technology, University of Technology Sydney, Ultimo, NSW 2007 Australia

Correction to: *Scientific Reports* https://doi.org/10.1038/s41598-022-16498-4, published online 20 July 2022

The original version of this Article contained an error in the spelling of the author Nima Darabi which was incorrectly given as Nima Darabai.

The Article also contained an error in the Equation in the Analyses based on competitions on evolutionary computation (CEC) section, under the subheading ‘Computational time and complexity analyses’ where “$O\left(FuFiO\right)$” was incorrectly given as “$O\left(FFO\right)$”.

$$\begin{aligned}O\left(FFO\right)&=O\left(t\times \left[O\left(sort\right)+O\left(nuclear\, reaction\, level\right)\right]\right)\\&=O\left(t\times \left[{n}^{2}+n\times d\right]\right)\\&=O(t{n}^{2}+nd)\end{aligned}$$

now reads:

$$ \begin{aligned} O\left( {FuFiO} \right) & = O\left( {t \times \left[ {O\left( {sort} \right) + O\left( {nuclear\;reaction\;level} \right)} \right]} \right) \\ & = O\left( {t \times \left[ {n^{2} + n \times d} \right]} \right) \\ \end{aligned} $$

In addition, Eq. 11 was incorrect,

$${X}_{i} = \left\{\begin{array}{c}{X}_{i}\, f\left({X}_{i}\right)\, is \, better \, than \, f\left({X}_{i}^{new}\right)\\ {X}_{i}^{new} f\left({X}_{i \, new}\right)\, is \, better \, than \, f\left({X}_{i}\right)\end{array}\right.$$

now reads:

$${X_i} = \left\{ \begin{aligned}
   & {X_i}\quad \;f\left( {{X_i}} \right)\;{\text{is}}\;{\text{better}}\;{\text{than}}\;f\left( {X_i^{new}} \right) \\ 
   & X_i^{new}\;f\left( {{X_{i\;new}}} \right)\;{\text{is}}\;{\text{better}}\;{\text{than}}\;f\left( {{X_i}} \right) \\ \end{aligned} \right.$$

Furthermore, the article contained errors in Fig. 13 where label (a) was omitted and “FuFiO” was incorrectly given as “FFO” on the left Figure of panel (d).

**Figure 13:**
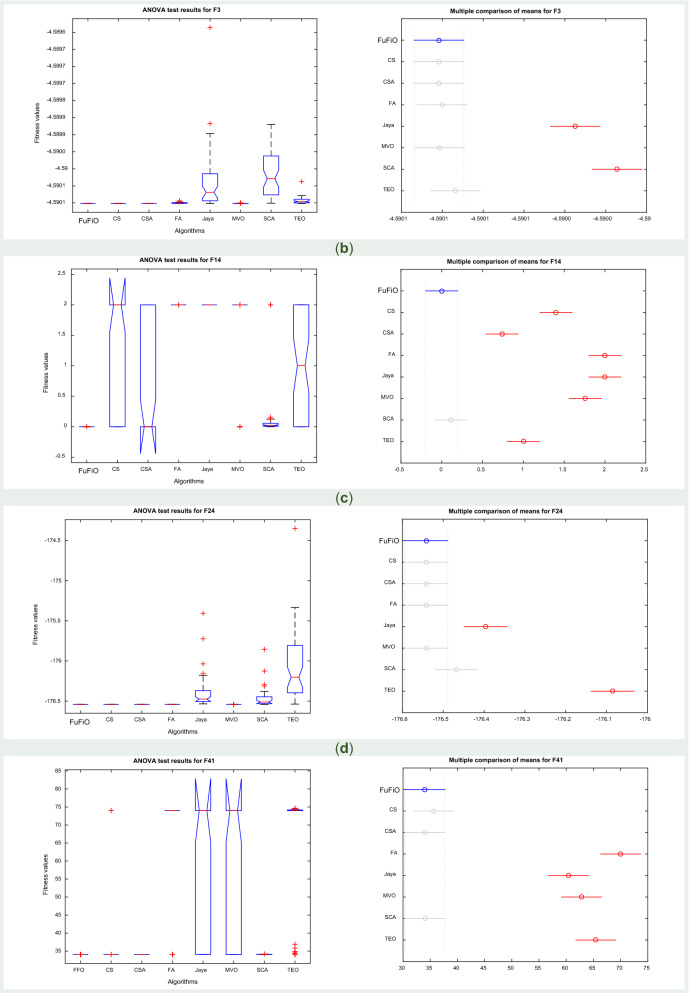
ANOVA test results for fixed-dimension functions.

The original Article has been corrected.

